# Supplementary material for: Genetic variability in microbial eukaryotes reshapes marine biodiversity assessment in the age of amplicon sequencing
Source: PLoS One. 2025 Jun 20;20(6):e0326053. doi: 10.1371/journal.pone.0326053 (PMC12180732; doi:10.1371/journal.pone.0326053)

Specimen:
Acrosphaera-murrayanna-01

Order: Collodaria

Cruise: P2
Net tow#:3

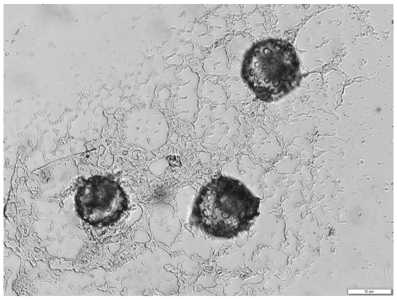

Specimen:
Acrosphaera-murrayanna-02

Order: Collodaria

Cruise: P2
Net tow#:4

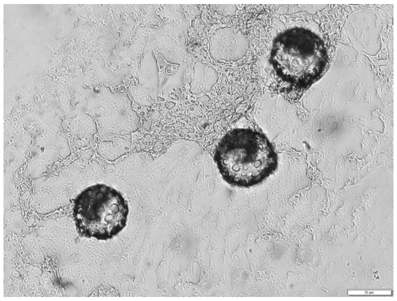

Specimen:
Acrosphaera-murrayanna-03

Order: Collodaria

Cruise: P2
Net tow#:4

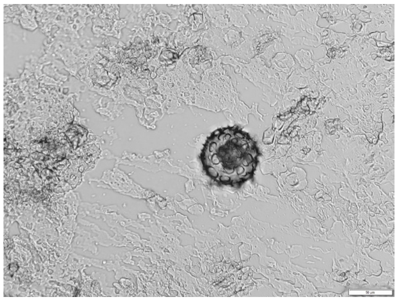

Specimen:
Acrosphaera-murrayanna-04

Order: Collodaria

Cruise: P2
Net tow#:4

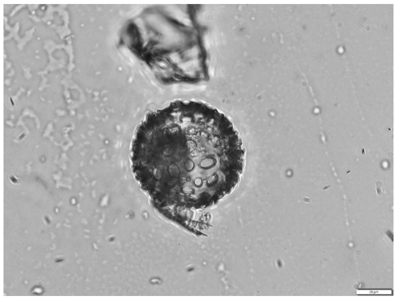

Specimen:
Acrosphaera-murrayanna-05

Order: Collodaria

Cruise: P2
Net tow#:4

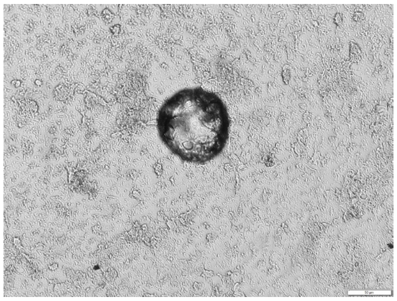

Specimen:
Acrosphaera-murrayanna-06

Order: Collodaria

Cruise: P2
Net tow#:4

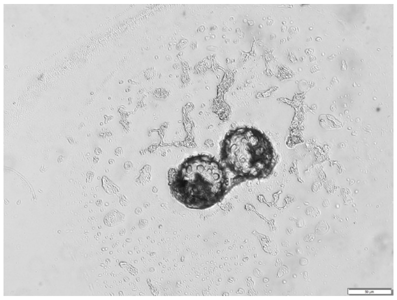

Specimen:
Collosphaera-huxleyi-01

Order: Collodaria

Cruise: HOT 338
Net tow#:3

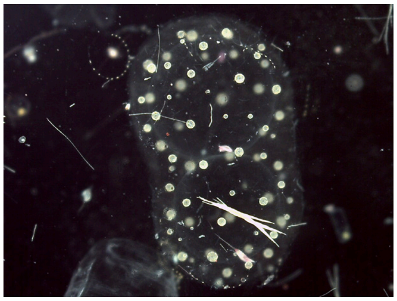

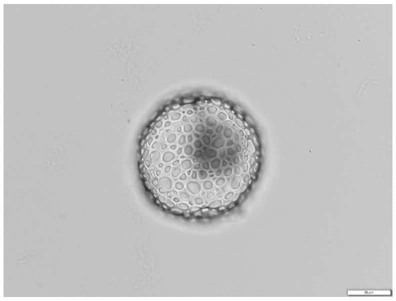

Specimen:
Collosphaera-huxleyi-02

Order: Collodaria

Cruise: HOT 338
Net tow#:3

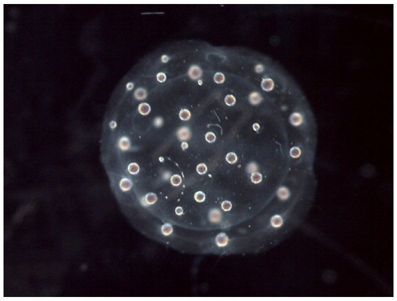

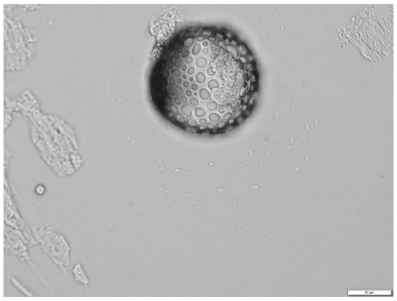

Specimen:
Collosphaera-huxleyi-03

Order: Collodaria

Cruise: HOT 338
Net tow#:5

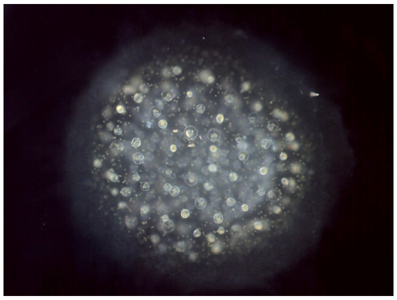

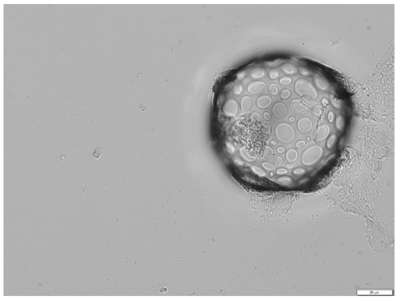

Specimen:
Collosphaera-huxleyi-04

Order: Collodaria

Cruise: HOT 338
Net tow#:5

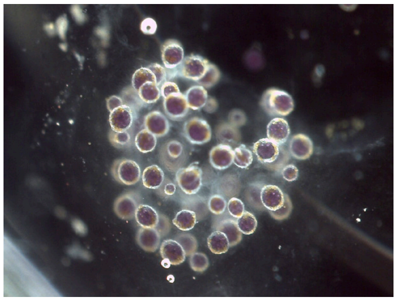

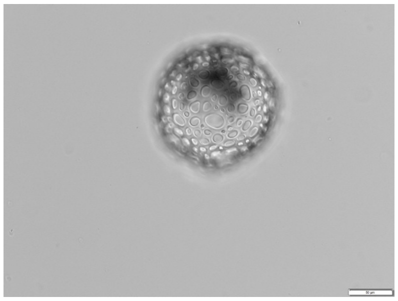

Specimen:
Collosphaera-huxleyi-05

Order: Collodaria

Cruise: HOT 338
Net tow#:6

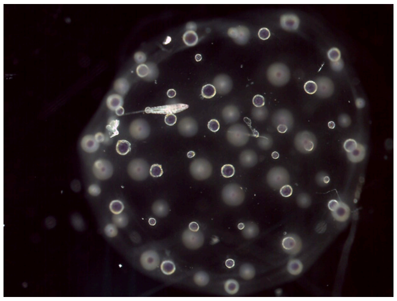

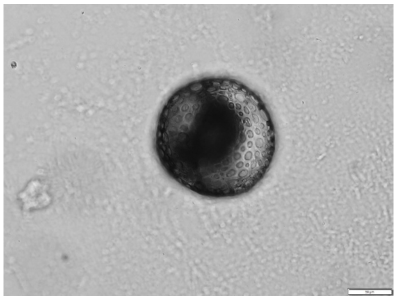

Specimen:
Collosphaera-huxleyi-06

Order: Collodaria

Cruise: HOT 339
Net tow#:11

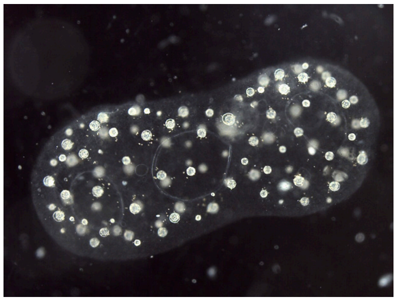

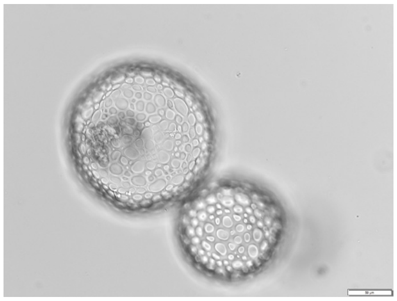

Specimen:
Collosphaera-huxleyi-07

Order: Collodaria

Cruise: HOT 339
Net tow#:11

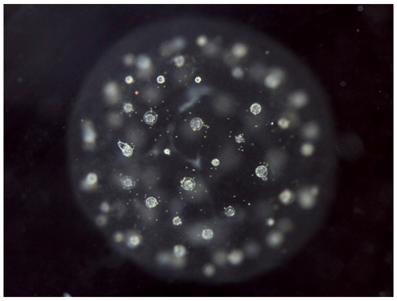

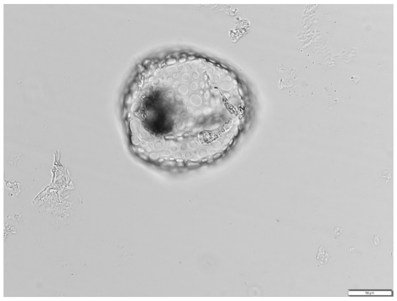

Specimen:
Collosphaera-huxleyi-08

Order: Collodaria

Cruise: HOT 339
Net tow#:11

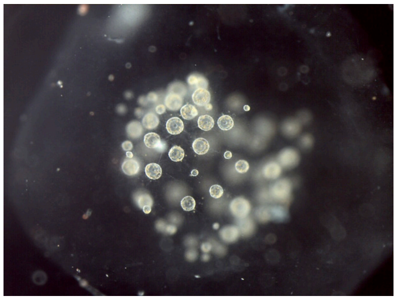

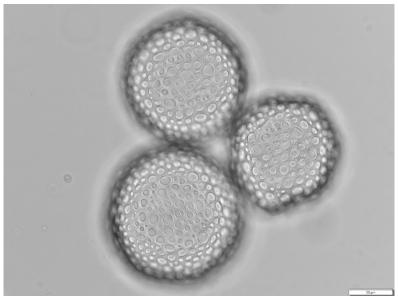

Specimen:
Collosphaera-huxleyi-09

Order: Collodaria

Cruise: HOT 339
Net tow#:11

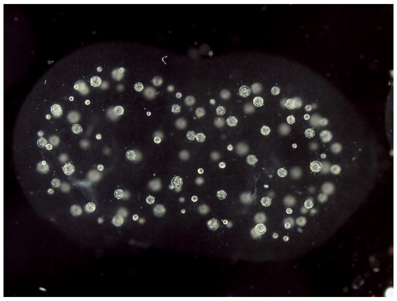

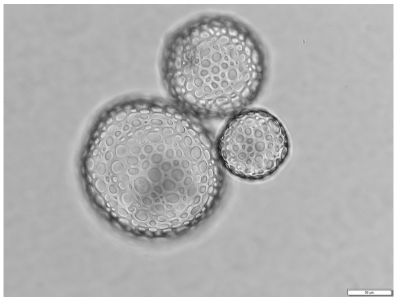

Specimen:
Collosphaera-huxleyi-10

Order: Collodaria

Cruise: HOT 339
Net tow#:11

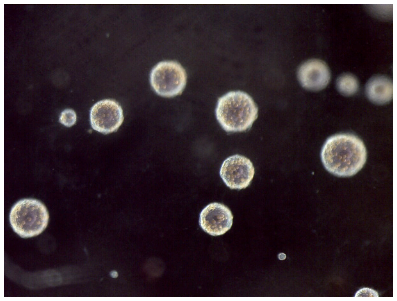

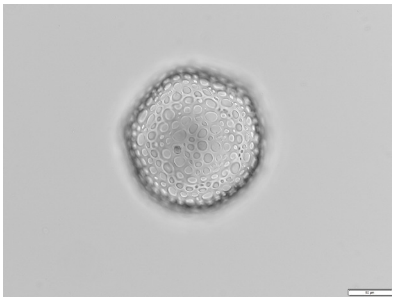

Specimen:
Collosphaera-huxleyi-11

Order: Collodaria

Cruise: HOT 339
Net tow#:11

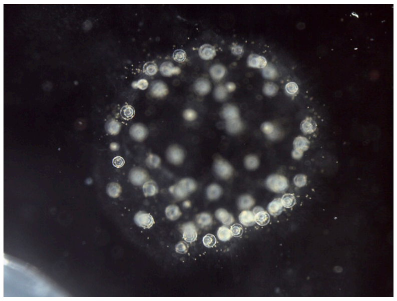

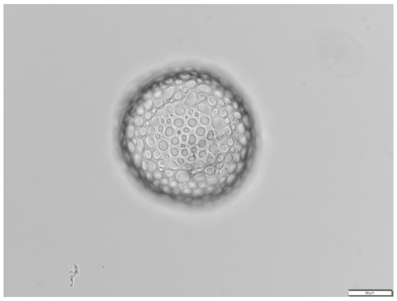

Specimen:
Collosphaera-huxleyi-12

Order: Collodaria

Cruise: HOT 339
Net tow#:11

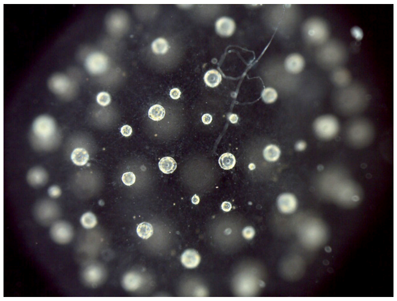

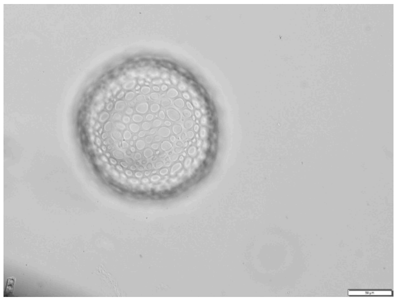

Specimen:
Collosphaera-huxleyi-13

Order: Collodaria

Cruise: HOT 339
Net tow#:17

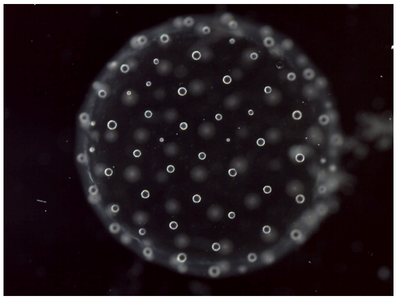

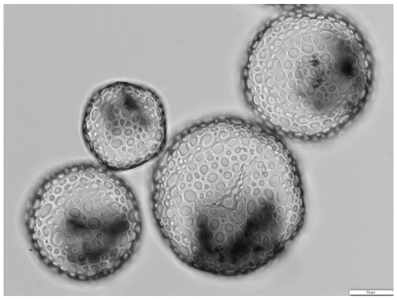

Specimen:
Collosphaera-huxleyi-14

Order: Collodaria

Cruise: HOT 339
Net tow#:17

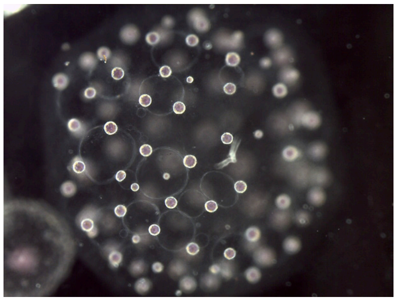

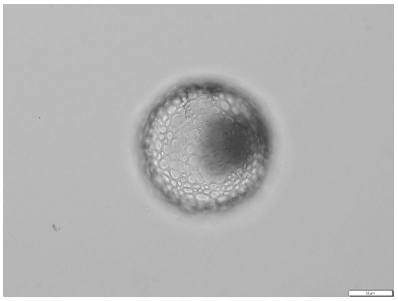

Specimen:
Collosphaera-huxleyi-15

Order: Collodaria

Cruise: HOT 339
Net tow#:17

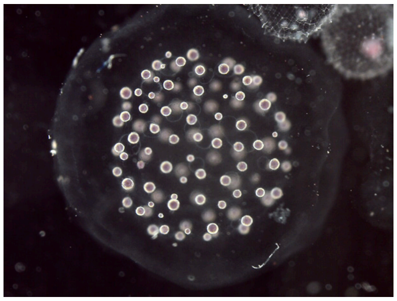

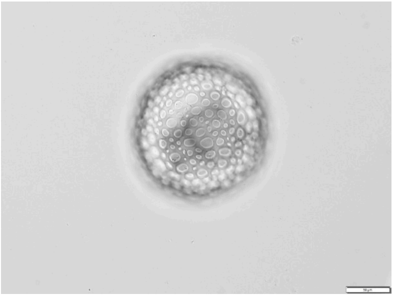

Specimen:
Collosphaera-huxleyi-16

Order: Collodaria

Cruise: HOT 339
Net tow#:17

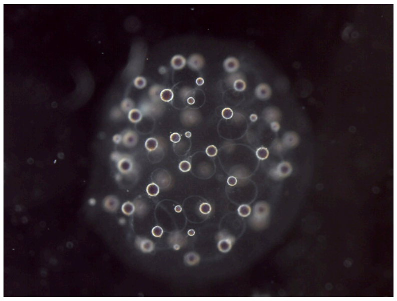

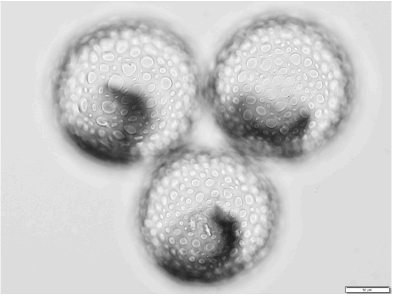

Specimen:
Collosphaera-huxleyi-17

Order: Collodaria

Cruise: HOT 339
Net tow#:17

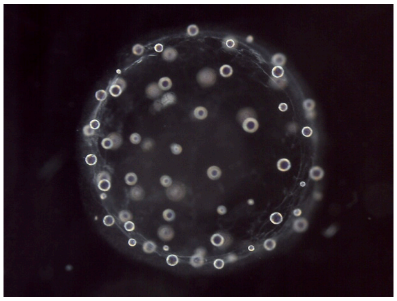

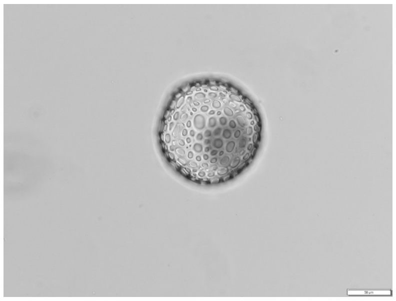

Specimen:
Collosphaera-huxleyi-18

Order: Collodaria

Cruise: HOT 339
Net tow#:5

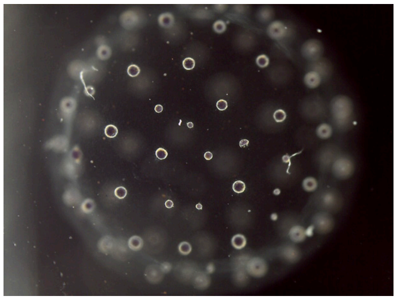

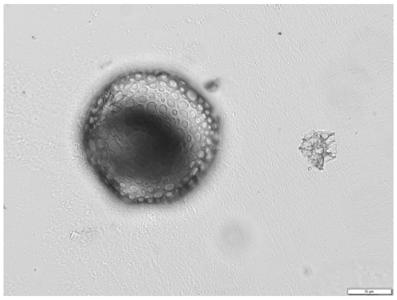

Specimen:
Collosphaera-huxleyi-19

Order: Collodaria

Cruise: HOT 339
Net tow#:5

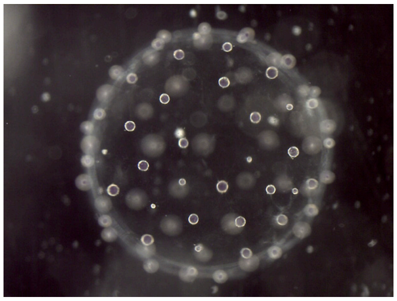

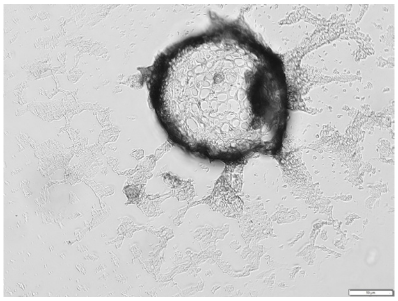

Specimen:
Collosphaera-huxleyi-20

Order: Collodaria

Cruise: HOT 339
Net tow#:5

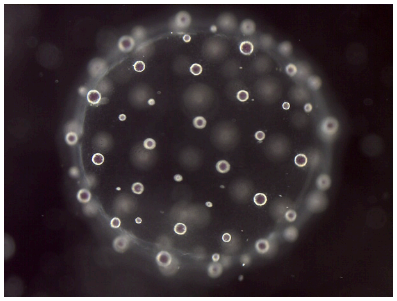

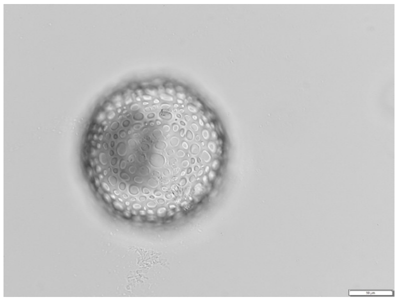

Specimen:
Collosphaera-huxleyi-21

Order: Collodaria

Cruise: HOT 339
Net tow#:5

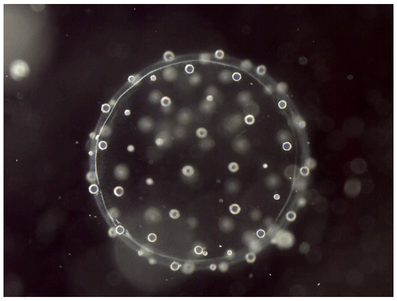

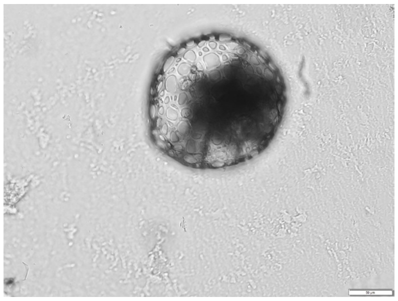

Specimen:
Collosphaera-huxleyi-22

Order: Collodaria

Cruise: HOT 339
Net tow#:5

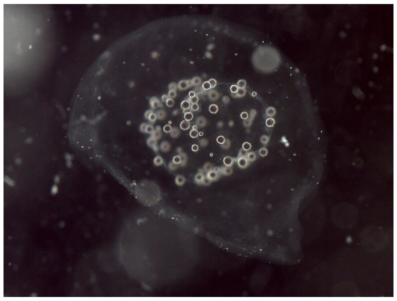

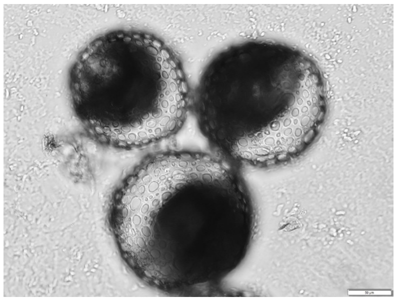

Specimen:
Collosphaera-huxleyi-23

Order: Collodaria

Cruise: HOT 339
Net tow#:5

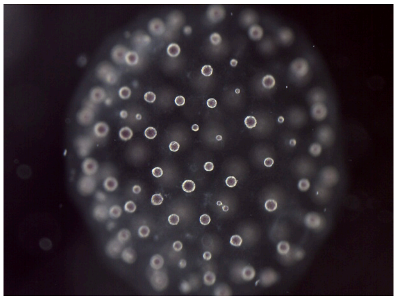

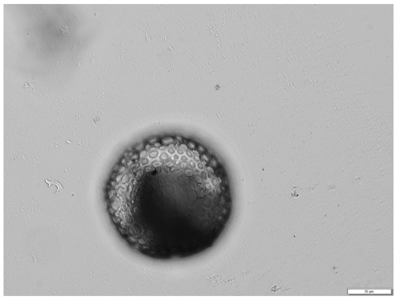

Specimen:
Collosphaera-huxleyi-24

Order: Collodaria

Cruise: HOT 339
Net tow#:5

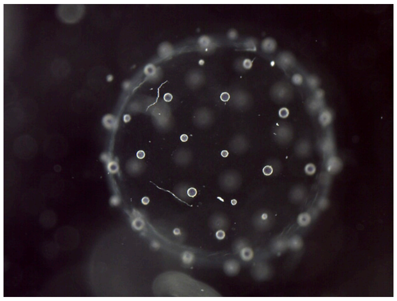

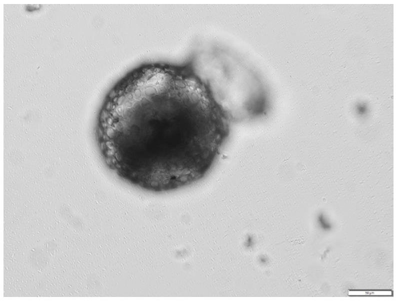

Specimen:
Collosphaera-huxleyi-25

Order: Collodaria

Cruise: HOT 339
Net tow#:5

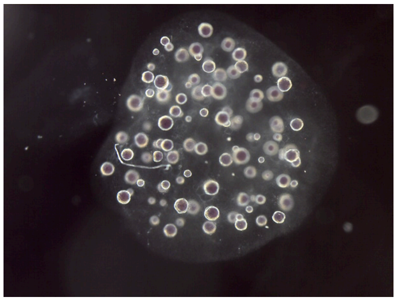
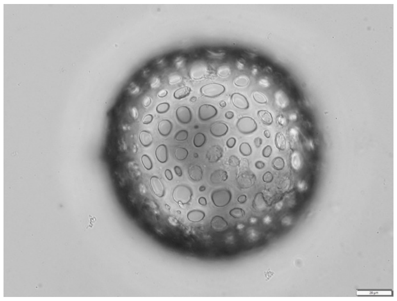

Specimen:
Collosphaera-huxleyi-26

Order: Collodaria

Cruise: HOT 339
Net tow#:5

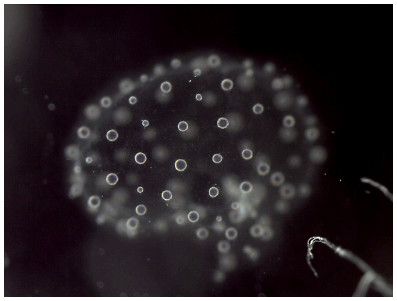
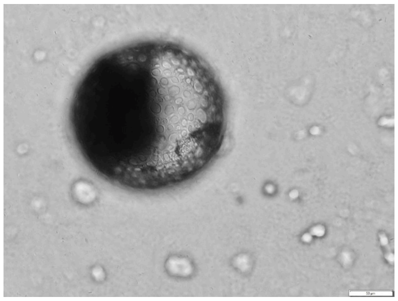

Specimen:
Collosphaera-huxleyi-27

Order: Collodaria

Cruise: HOT 339
Net tow#:5

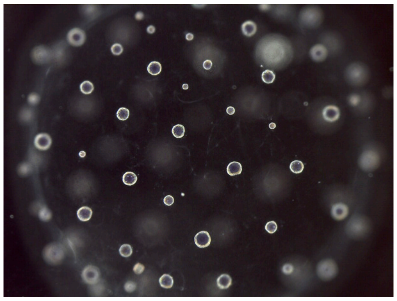
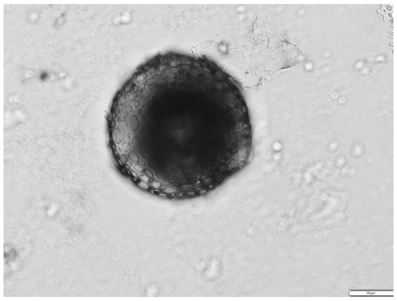

Specimen:
Collosphaera-huxleyi-28

Order: Collodaria

Cruise: P2
Net tow#:1

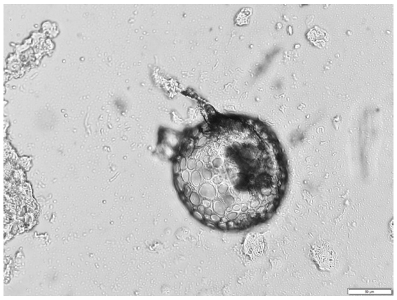

Specimen:
Collosphaera-huxleyi-29

Order: Collodaria

Cruise: P2
Net tow#:1

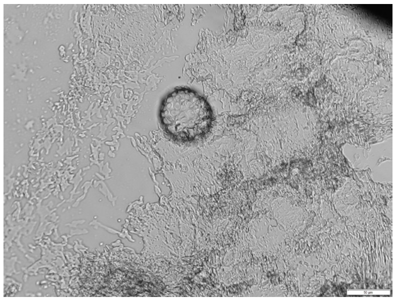

Specimen:
Collosphaera-huxleyi-30

Order: Collodaria

Cruise: P2
Net tow#:1

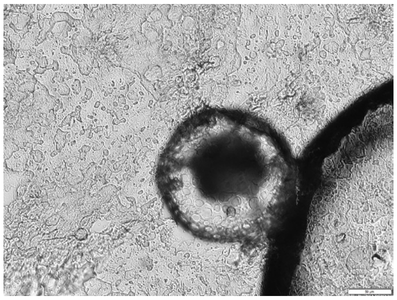

Specimen:
Collosphaera-huxleyi-31

Order: Collodaria

Cruise: P2
Net tow#:1

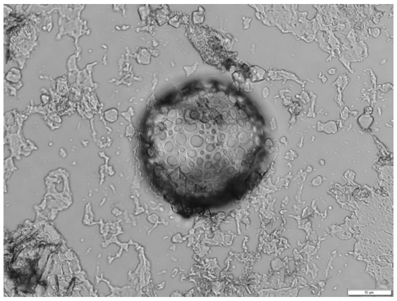

Specimen:
Collosphaera-huxleyi-32

Order: Collodaria

Cruise: P2
Net tow#:1

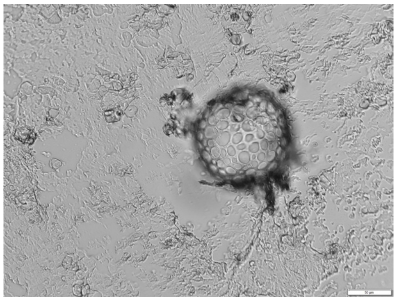

Specimen:
Collosphaera-huxleyi-33

Order: Collodaria

Cruise: P2
Net tow#:1

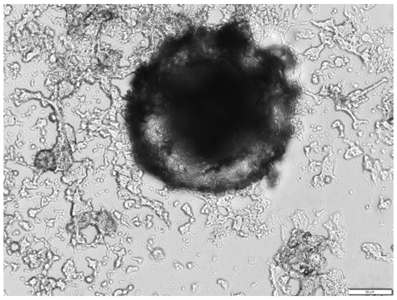

Specimen:
Collosphaera-huxleyi-34

Order: Collodaria

Cruise: P2
Net tow#:2

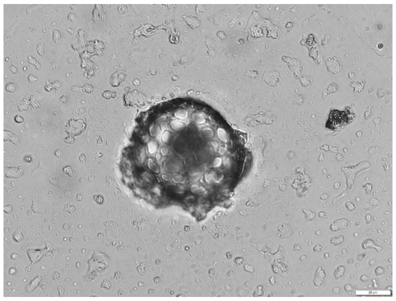

Specimen:
Collosphaera-huxleyi-35

Order: Collodaria

Cruise: P2
Net tow#:2

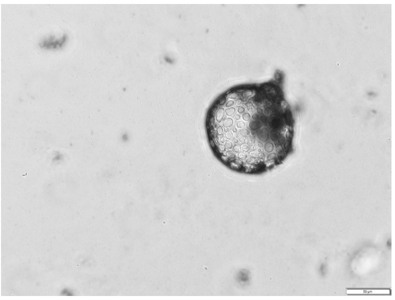

Specimen:
Collosphaera-huxleyi-36

Order: Collodaria

Cruise: P2
Net tow#:3

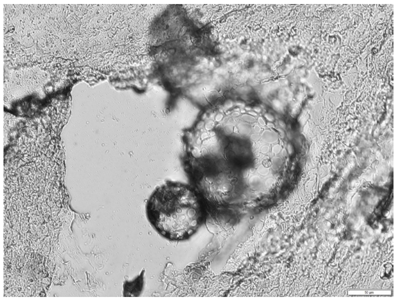

Specimen:
Collosphaera-huxleyi-37

Order: Collodaria

Cruise: P2
Net tow#:3

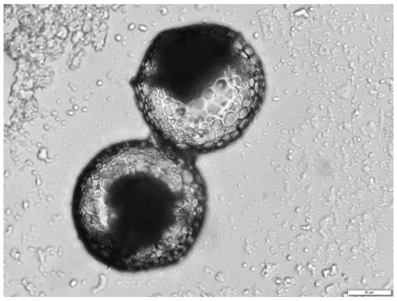

Specimen:
Collosphaera-huxleyi-38

Order: Collodaria

Cruise: P2
Net tow#:3

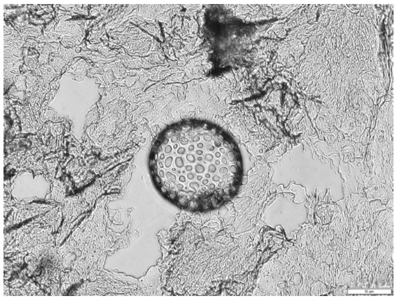

Specimen:
Collosphaera-huxleyi-39

Order: Collodaria

Cruise: P2
Net tow#:3

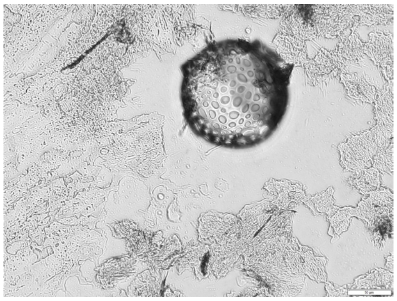

Specimen:
Collosphaera-huxleyi-40

Order: Collodaria

Cruise: P2
Net tow#:3

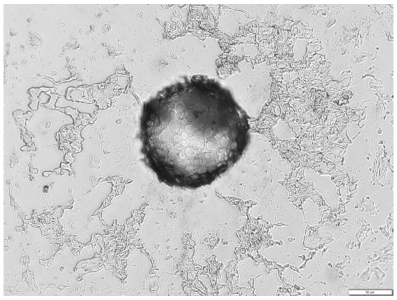

Specimen:
Collosphaera-huxleyi-41

Order: Collodaria

Cruise: P2
Net tow#:4

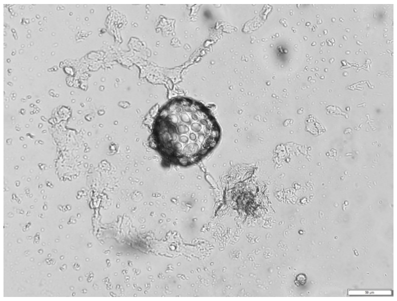

Specimen:
Collosphaera-huxleyi-42

Order: Collodaria

Cruise: P2
Net tow#:4

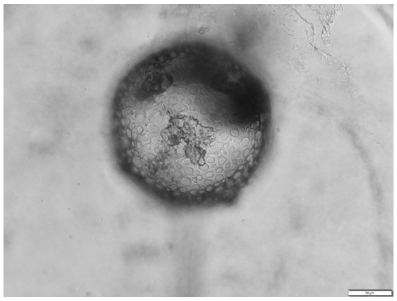

Specimen:
Collosphaera-huxleyi-43

Order: Collodaria

Cruise: P2
Net tow#:4

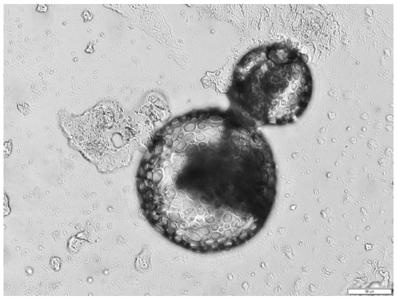

Specimen:
Collosphaera-huxleyi-44

Order: Collodaria

Cruise: P2
Net tow#:4

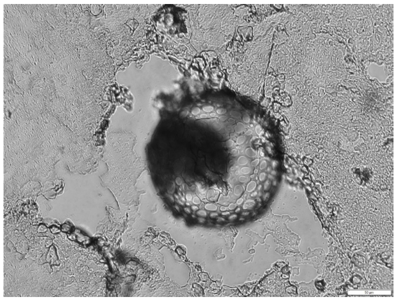

Specimen:
Collosphaera-huxleyi-45

Order: Collodaria

Cruise: P2
Net tow#:4

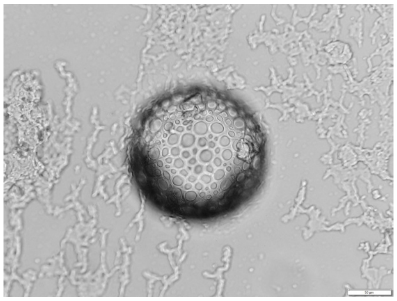

Specimen:
Collosphaera-huxleyi-46

Order: Collodaria

Cruise: P2
Net tow#:4

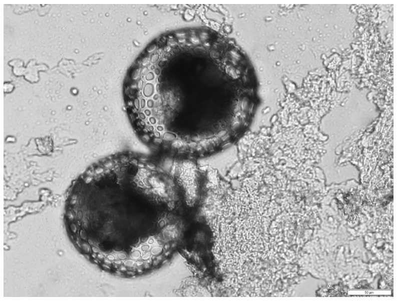

Specimen:
Collosphaera-huxleyi-47

Order: Collodaria

Cruise: P2
Net tow#:4

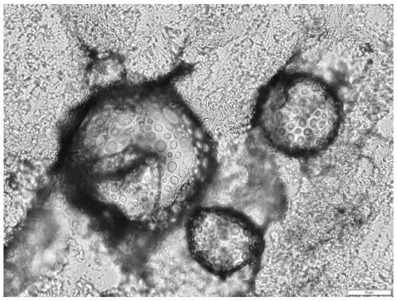

Specimen:
Collosphaera-huxleyi-48

Order: Collodaria

Cruise: P2
Net tow#:4

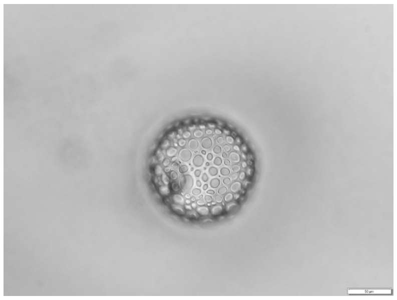

Specimen:
Collosphaera-huxleyi-49

Order: Collodaria

Cruise: P2
Net tow#:4

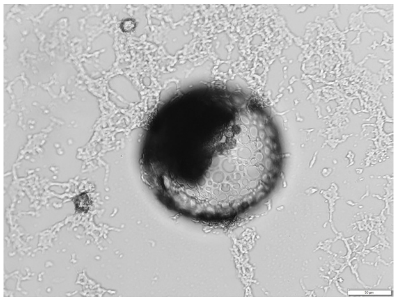

Specimen:
Collosphaera-huxleyi-50

Order: Collodaria

Cruise: P2
Net tow#:4

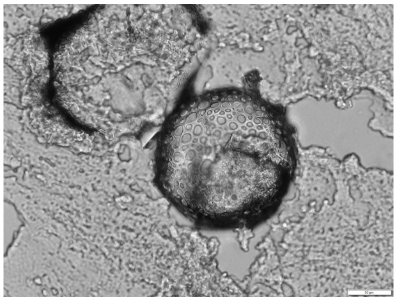

Specimen:
Collosphaera-huxleyi-51

Order: Collodaria

Cruise: P2
Net tow#:4

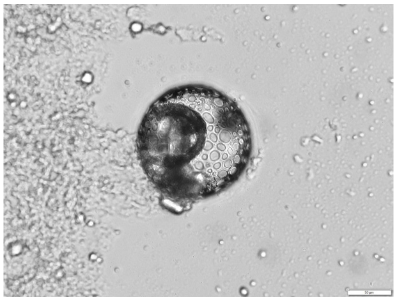

Specimen:
Disolenia-zanguebarica-01

Order: Collodaria

Cruise: P2
Net tow#:3

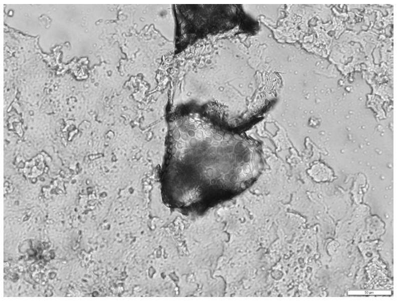

Specimen:
Disolenia-zanguebarica-02

Order: Collodaria

Cruise: P2
Net tow#:4

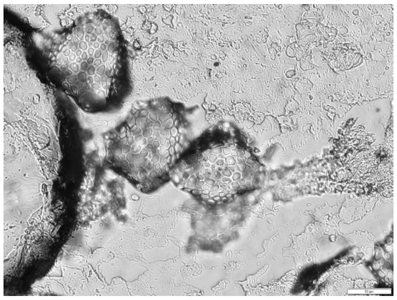

Specimen:
Siphonosphaera-socialis-colony-01

Order: Collodaria

Cruise: HOT 339
Net tow#:15

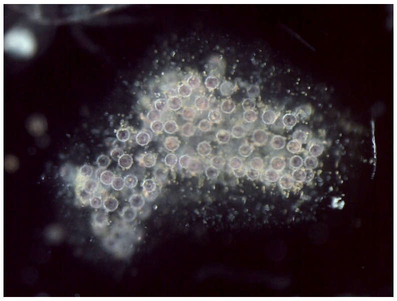

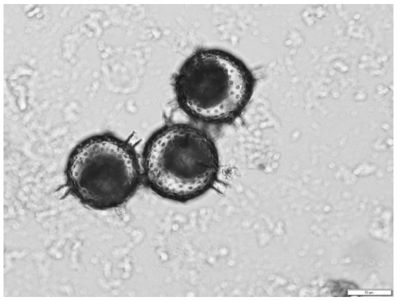

Specimen:
Siphonosphaera-socialis-colony-02

Order: Collodaria

Cruise: HOT 338
Net tow#:5

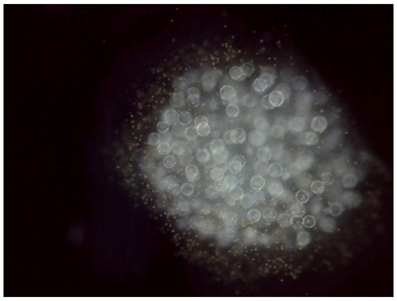

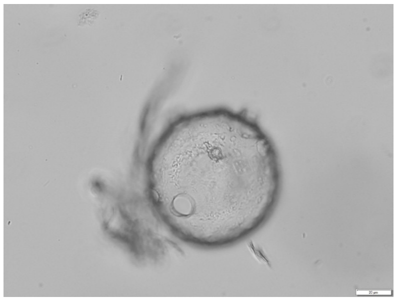

Specimen:
Siphonosphaera-socialis-colony-03

Order: Collodaria

Cruise: P2
Net tow#:1

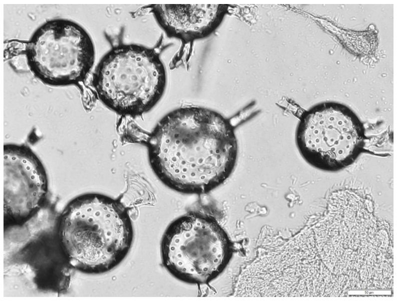

Specimen:
Siphonosphaera-socialis-single-cell-01

Order: Collodaria

Cruise: HOT 339
Net tow#:15

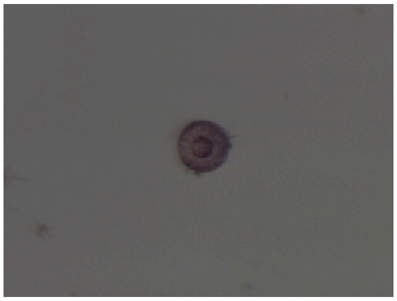

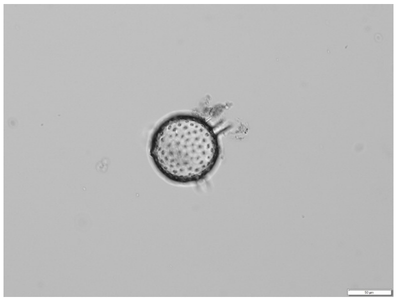

Specimen:
Siphonosphaera-socialis-single-cell-02

Order: Collodaria

Cruise: HOT 338
Net tow#:9

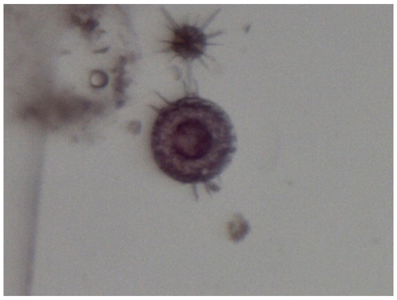

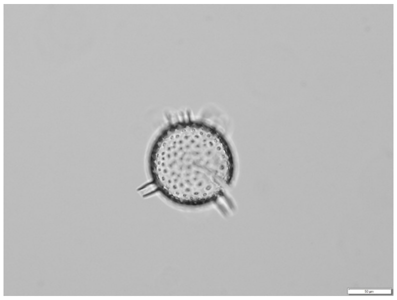

Specimen:
Siphonosphaera-socialis-single-cell-03

Order: Collodaria

Cruise: HOT 338
Net tow#:9

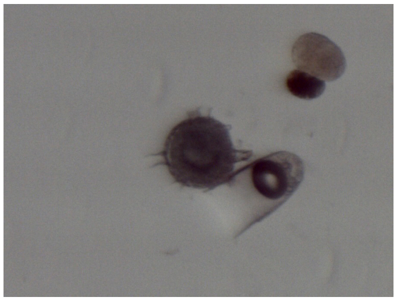

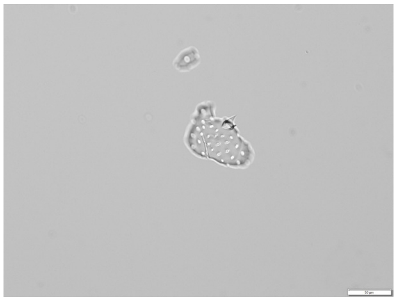

Specimen:
Collozoum-sp-01

Order: Collodaria

Cruise: HOT 338
Net tow#:5

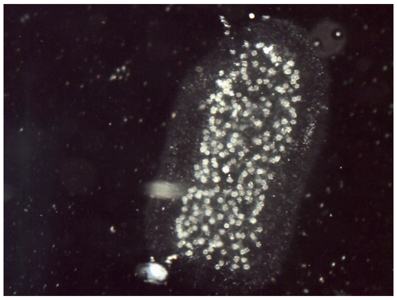

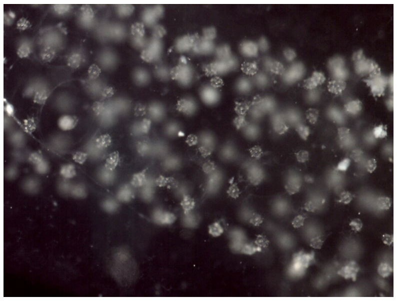

Specimen:
Collozoum-sp-02

Order: Collodaria

Cruise: HOT 339
Net tow#:11

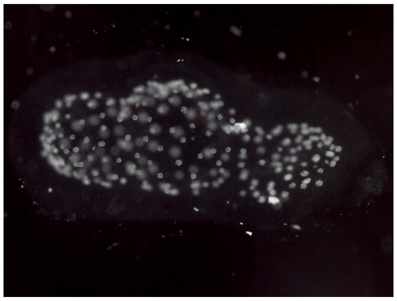

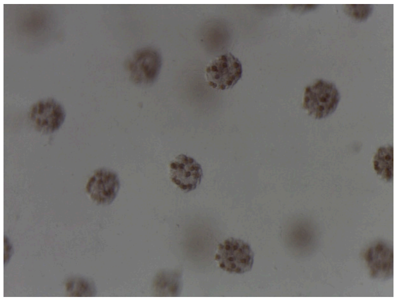

Specimen:
Collozoum-sp-03

Order: Collodaria

Cruise: HOT 339
Net tow#:17

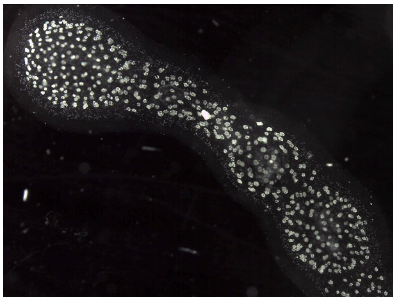

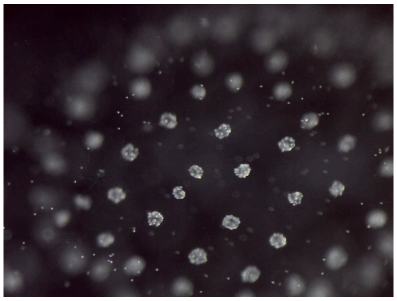

Specimen:
Collozoum-sp-04

Order: Collodaria

Cruise: HOT 339
Net tow#:17

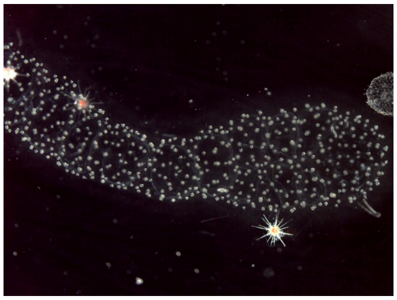

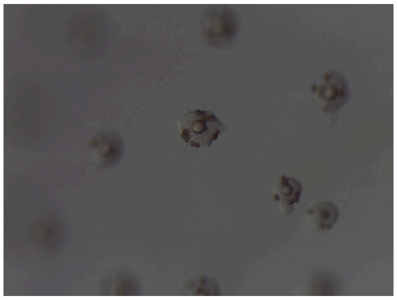

Specimen:
Collozoum-sp-05

Order: Collodaria

Cruise: HOT 339
Net tow#:17

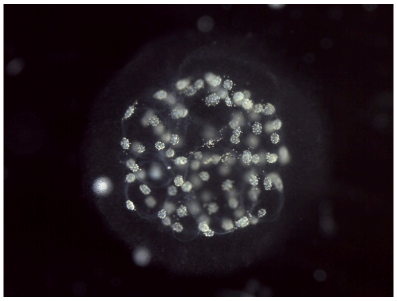

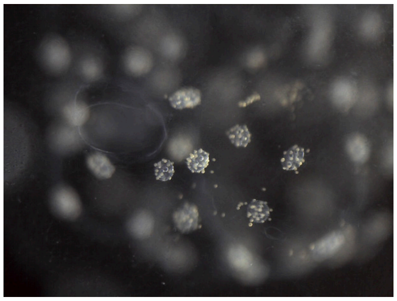

Specimen:
Collozoum-sp-06

Order: Collodaria

Cruise: HOT 339
Net tow#:11

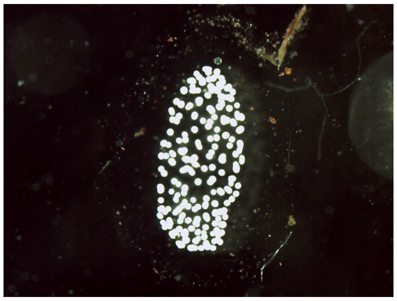

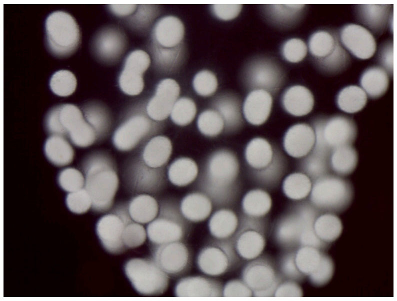

Specimen:
Procyttarium-primordialis?-01

Order: Collodaria

Cruise: HOT 339
Net tow#:8

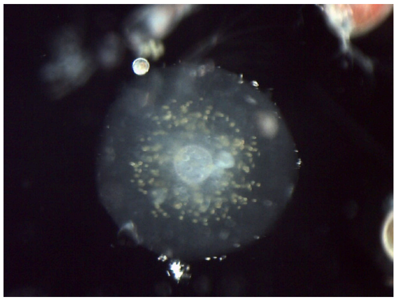

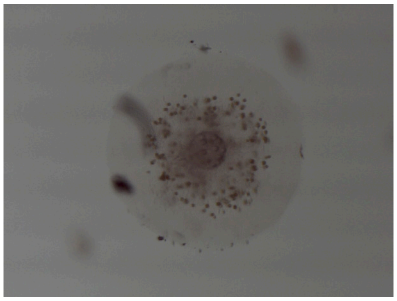

Specimen:
Procyttarium-primordialis?-02

Order: Collodaria

Cruise: HOT 339
Net tow#:11

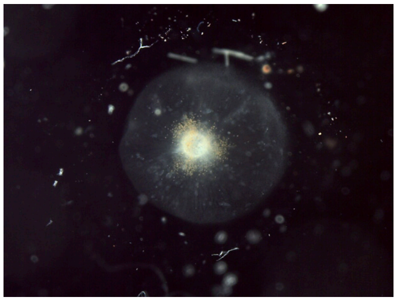

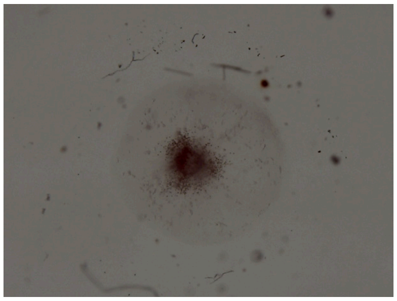

Specimen:
Procyttarium-primordialis?-03

Order: Collodaria

Cruise: HOT 339
Net tow#:14

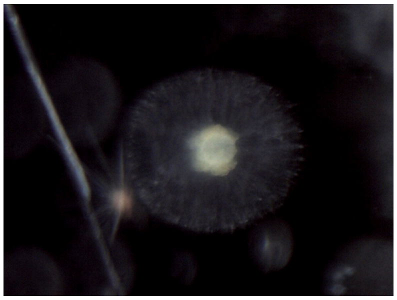

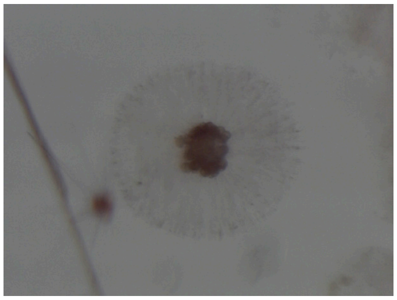

Specimen:
Rhaphidozoum-sp-01

Order: Collodaria

Cruise: HOT 339
Net tow#:11

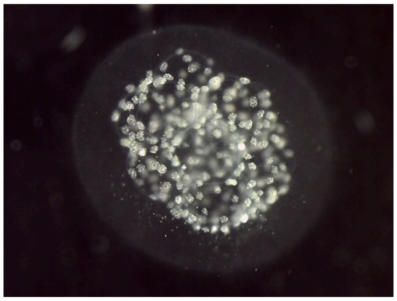

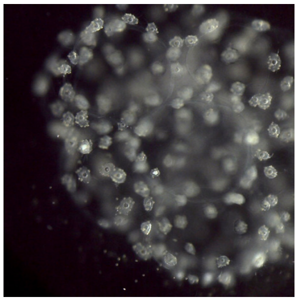

Specimen:
Rhaphidozoum-sp-02

Order: Collodaria

Cruise: HOT 339
Net tow#:17

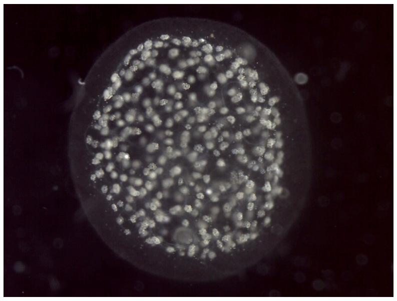

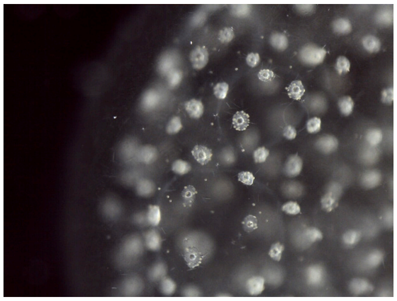

Specimen:
Rhaphidozoum-sp-03

Order: Collodaria

Cruise: HOT 339
Net tow#:17

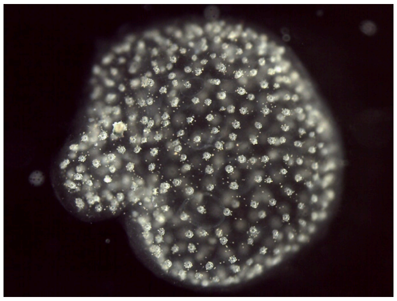

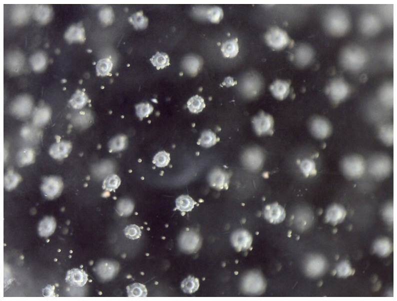

Specimen:
Sphaerozoum-sp-01

Order: Collodaria

Cruise: HOT 339
Net tow#:11

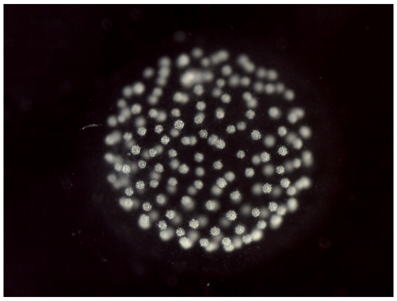

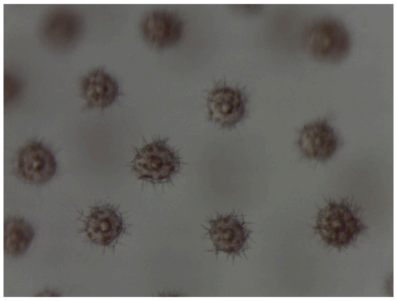

Specimen:
Sphaerozoum-sp-02

Order: Collodaria

Cruise: HOT 339
Net tow#:5

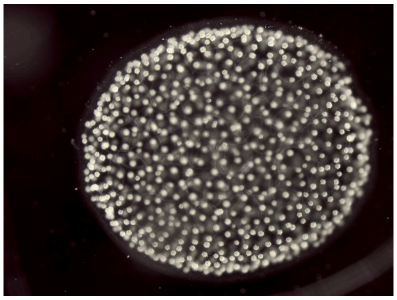

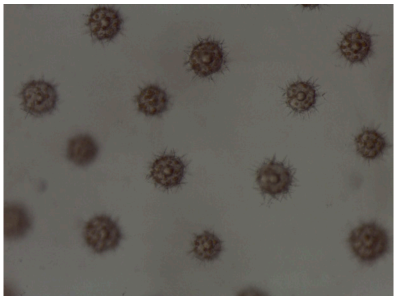

Specimen:
Sphaerozoum-sp-03

Order: Collodaria

Cruise: HOT 339
Net tow#:5

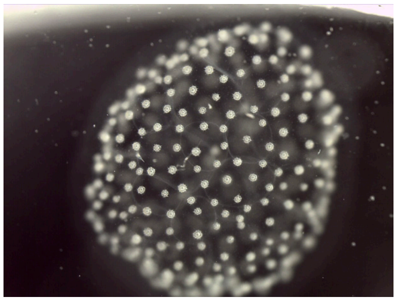

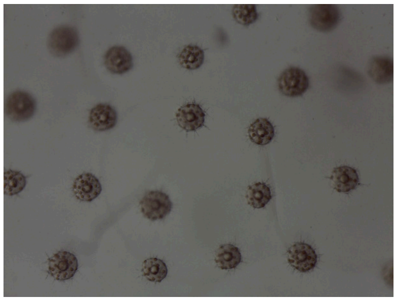

Specimen:
Sphaerozoum-sp-04

Order: Collodaria

Cruise: HOT 339
Net tow#:11

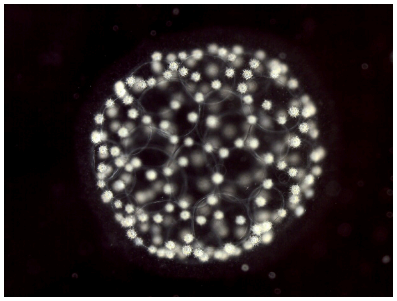

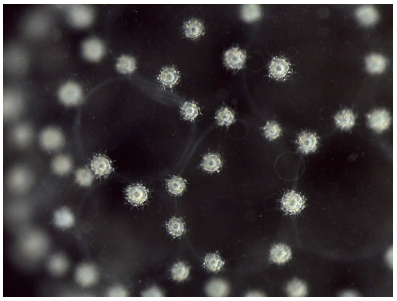

Specimen:
Sphaerozoum-sp-05

Order: Collodaria

Cruise: HOT 339
Net tow#:5

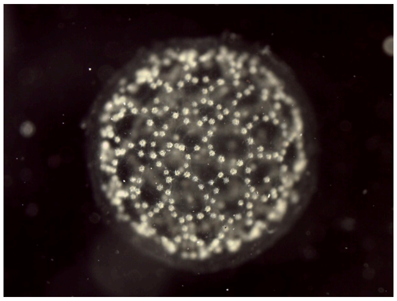

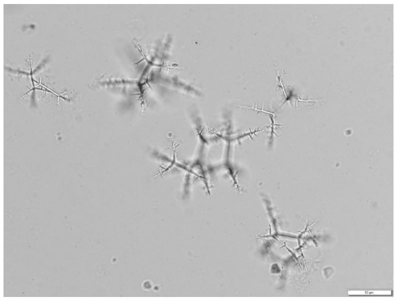

Specimen:
Sphaerozoum-sp-06

Order: Collodaria

Cruise: HOT 339
Net tow#:5

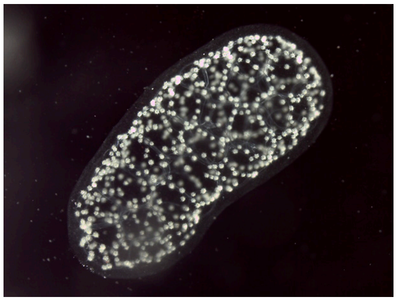

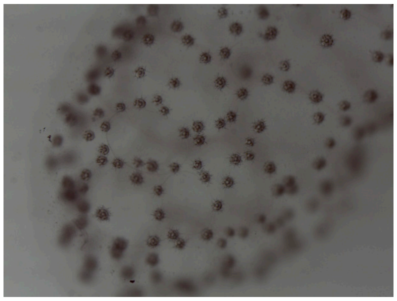

Specimen:
Sphaerozoum-sp-07

Order: Collodaria

Cruise: HOT 339
Net tow#:5

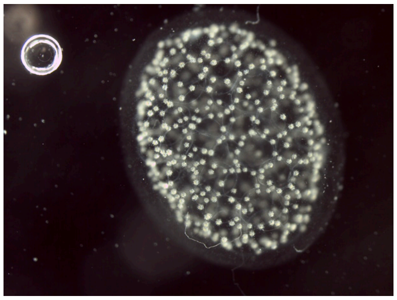

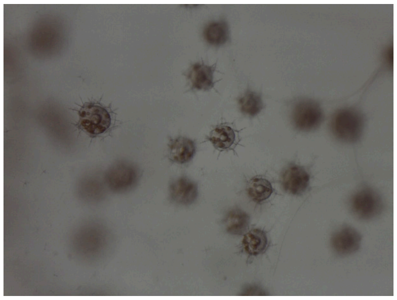

Specimen:
Sphaerozoum-sp-08

Order: Collodaria

Cruise: HOT 339
Net tow#:5

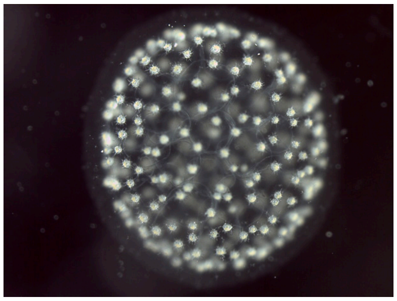

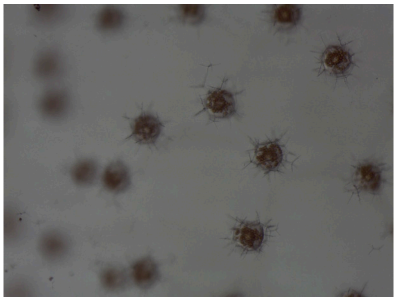

Specimen:
Thalassicolla-nucleata-01

Order: Collodaria

Cruise: HOT 339
Net tow#:11

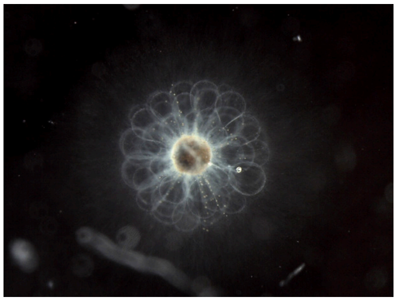

Specimen:
Thalassicolla-nucleata-02

Order: Collodaria

Cruise: HOT 339
Net tow#:11

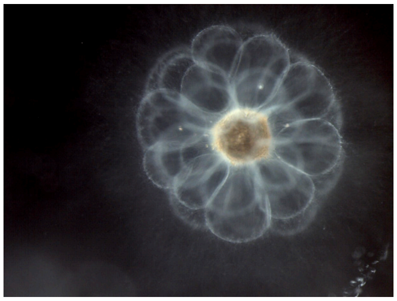

Specimen:
Thalassicolla-nucleata-03

Order: Collodaria

Cruise: HOT 339
Net tow#:11

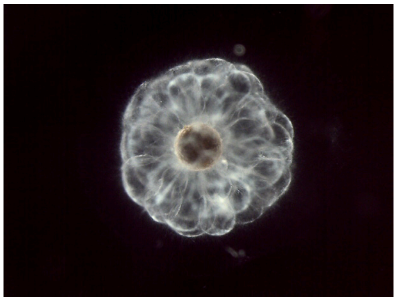

Specimen:
Thalassicolla-nucleata-04

Order: Collodaria

Cruise: HOT 339
Net tow#:11

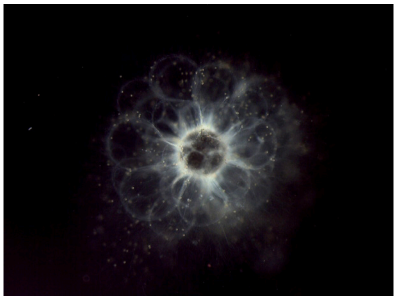

Specimen:
Thalassicolla-nucleata-05

Order: Collodaria

Cruise: HOT 339
Net tow#:5

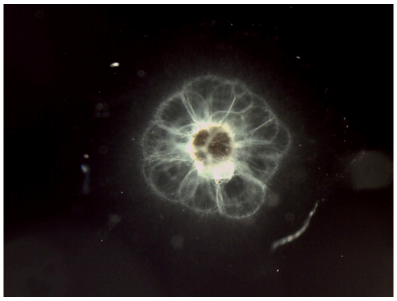

Specimen:
Thalassicolla-nucleata-06

Order: Collodaria

Cruise: HOT 339
Net tow#:5

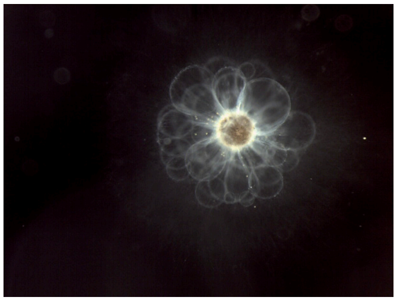

Specimen:
Thalassicolla-nucleata-07

Order: Collodaria

Cruise: HOT 339
Net tow#:5

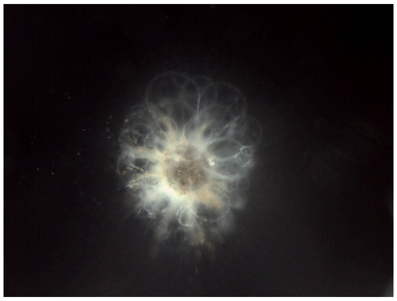

Specimen:
Thalassicolla-nucleata-08

Order: Collodaria

Cruise: HOT 339
Net tow#:5

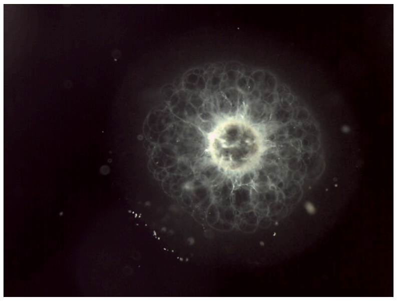

Specimen:
Thalassicolla-nucleata-09

Order: Collodaria

Cruise: HOT 339
Net tow#:5

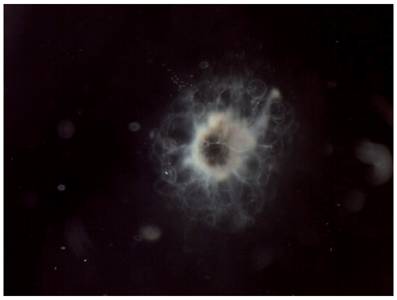

Specimen:
Thalassicolla-nucleata-10

Order: Collodaria

Cruise: HOT 339
Net tow#:5

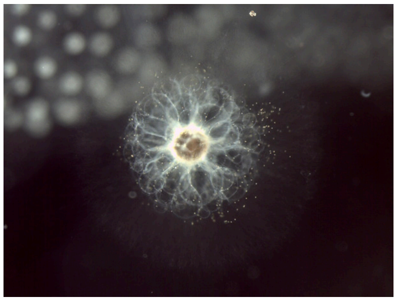

Specimen:
Acanthodesmia-vinculata-01

Order: Nassellaria

Cruise: HOT 338
Net tow#:7

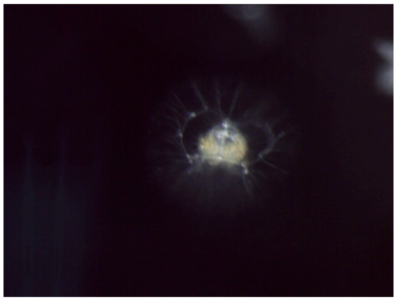

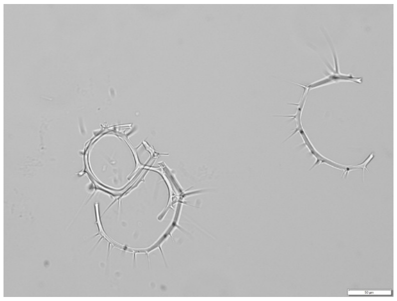

Specimen:
Acanthodesmia-vinculata-02

Order: Nassellaria

Cruise: HOT 339
Net tow#:15

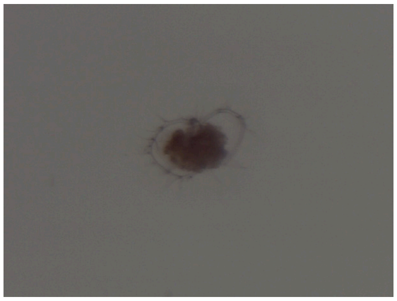

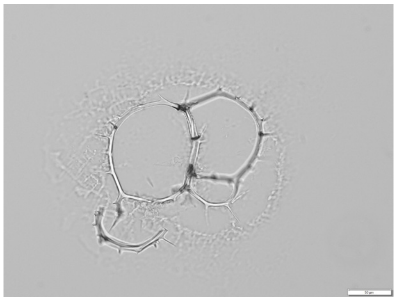

Specimen:
acanthodesmid-sp-01

Order: Nassellaria

Cruise: HOT 339
Net tow#:2

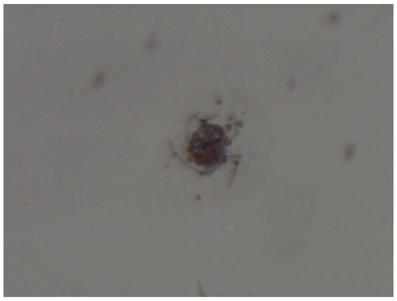

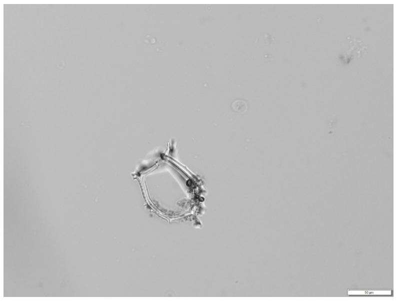

Specimen:
juvenile-acanthodesmid-01

Order: Nassellaria

Cruise: HOT 339
Net tow#:2

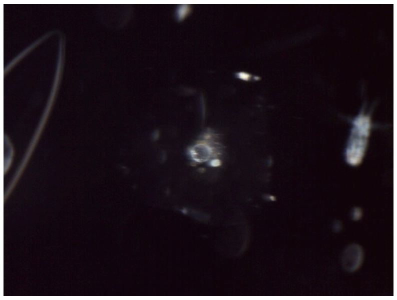

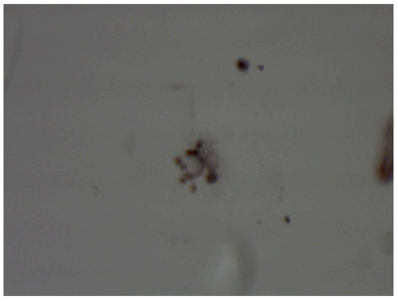

Specimen:
Eucyrtidium-hexagonatum-01

Order: Nassellaria

Cruise: HOT 339
Net tow#:19

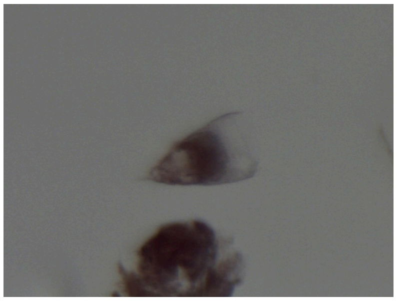

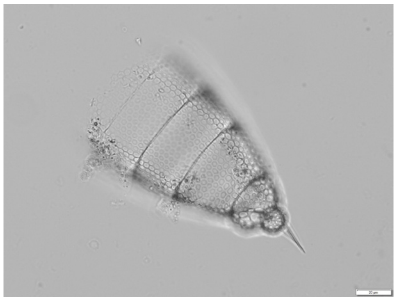

Specimen:
Eucyrtidium-hexagonatum-02

Order: Nassellaria

Cruise: HOT 339
Net tow#:2

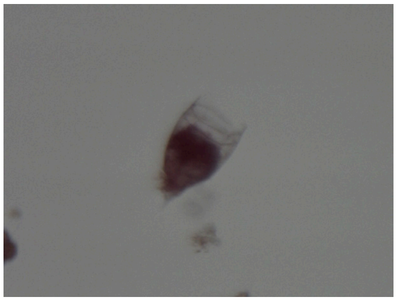

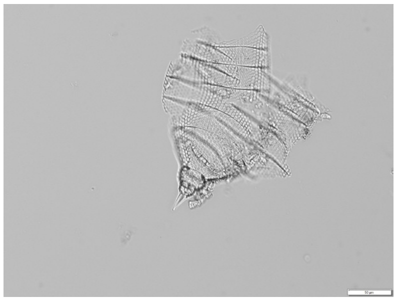

Specimen:
Lophophaena-hispida-01

Order: Nassellaria

Cruise: HOT 339
Net tow#:9

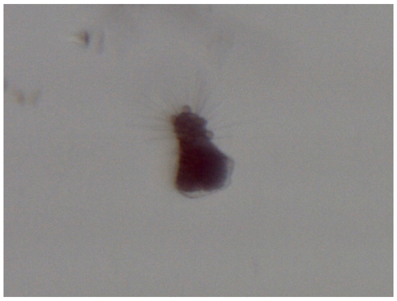

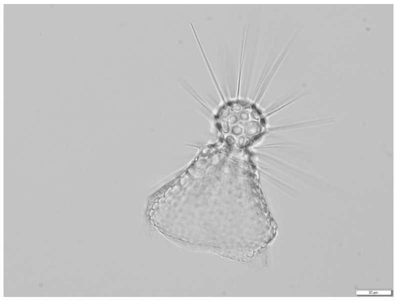

Specimen:
Peromelissa-thoracites-01

Order: Nassellaria

Cruise: HOT 339
Net tow#:10

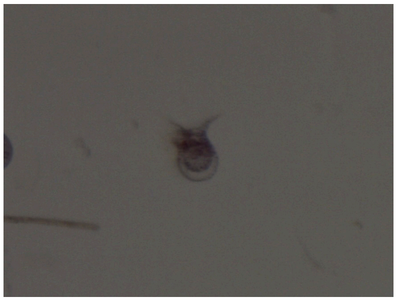

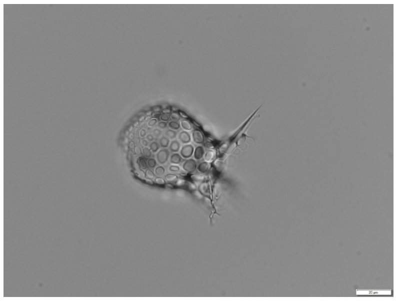

Specimen:
Pterocorys-campanula-01

Order: Nassellaria

Cruise: HOT 339
Net tow#:12

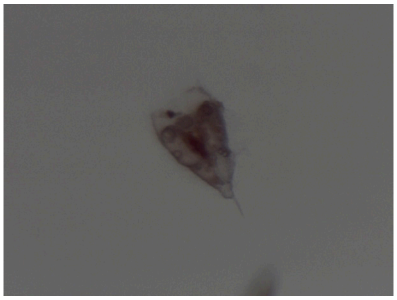

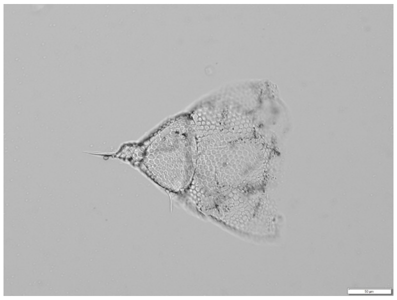

Specimen:
Pterocorys-sp-01

Order: Nassellaria

Cruise: HOT 339
Net tow#:10

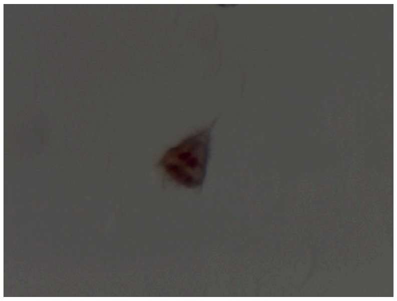

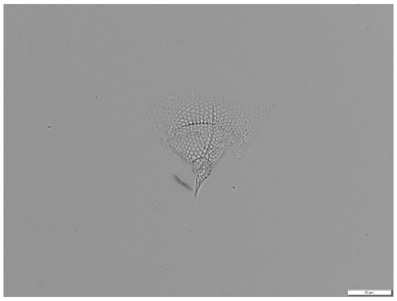

Specimen:
Pterocorys-sp-02

Order: Nassellaria

Cruise: HOT 339
Net tow#:10

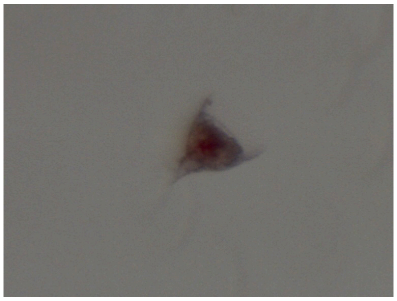

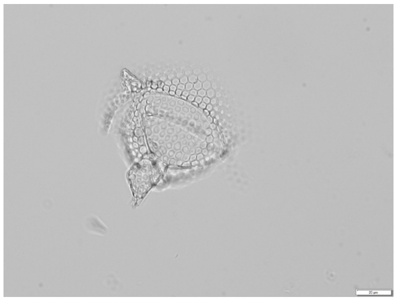

Specimen:
Pterocorys-sp-03

Order: Nassellaria

Cruise: HOT 339
Net tow#:15

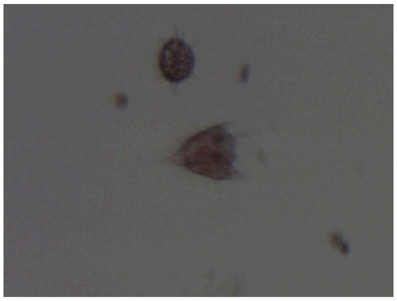

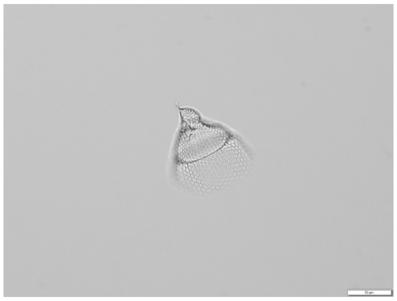

Specimen:
Pterocorys-sp-04

Order: Nassellaria

Cruise: HOT 339
Net tow#:19

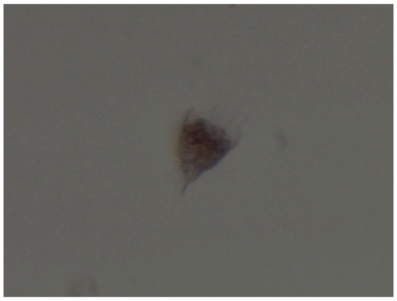

Specimen:
Pterocorys-zancleus-01

Order: Nassellaria

Cruise: HOT 339
Net tow#:10

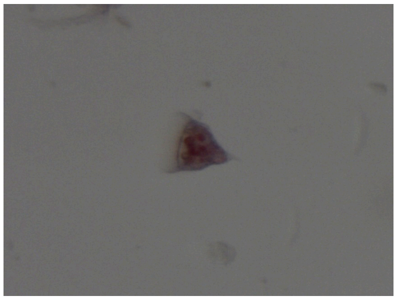

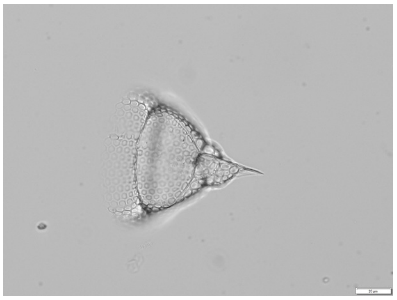

Specimen:
Pterocorys-zancleus-02

Order: Nassellaria

Cruise: HOT 339
Net tow#:10

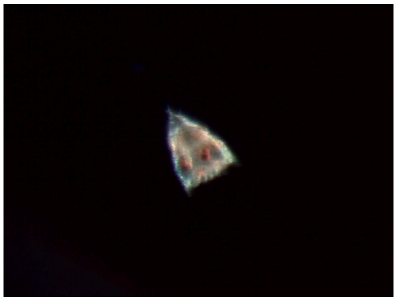

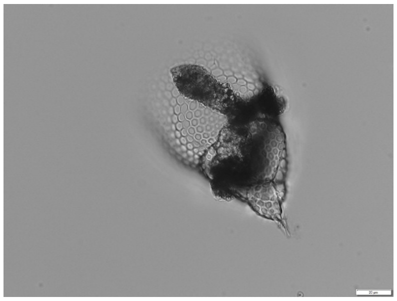

Specimen:
Pterocorys-zancleus-03

Order: Nassellaria

Cruise: HOT 339
Net tow#:10

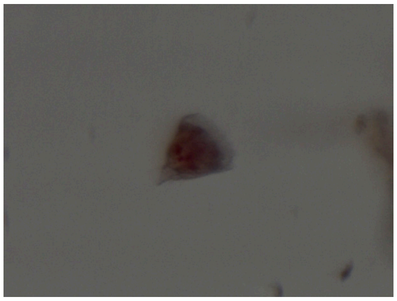

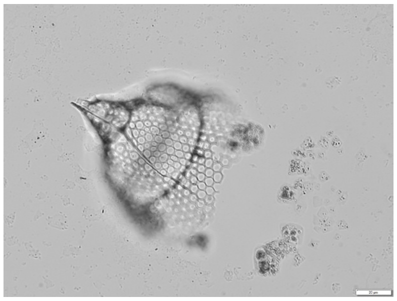

Specimen:
Pterocorys-zancleus-04

Order: Nassellaria

Cruise: HOT 339
Net tow#:10

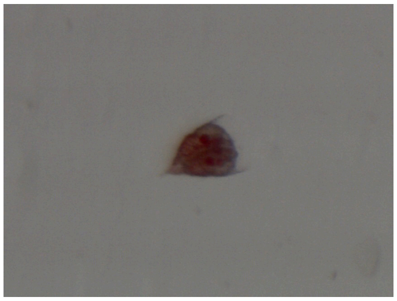

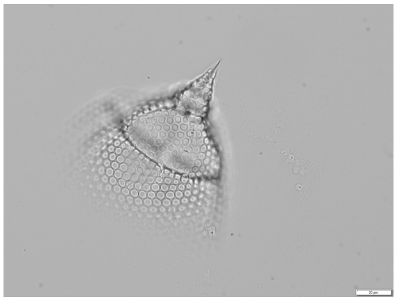

Specimen:
Pterocorys-zancleus-05

Order: Nassellaria

Cruise: HOT 339
Net tow#:13

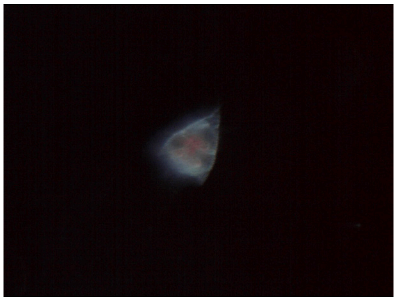

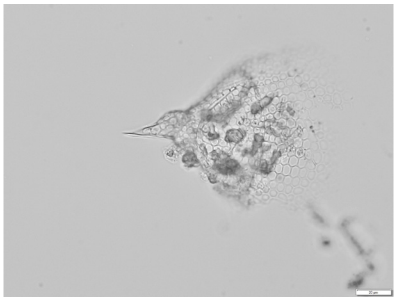

Specimen:
Pterocorys-zancleus-06

Order: Nassellaria

Cruise: HOT 339
Net tow#:19

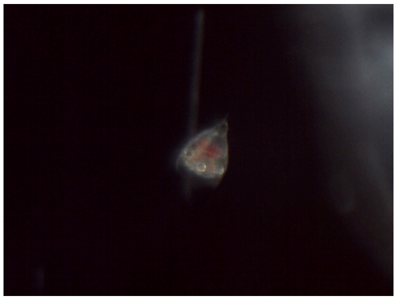

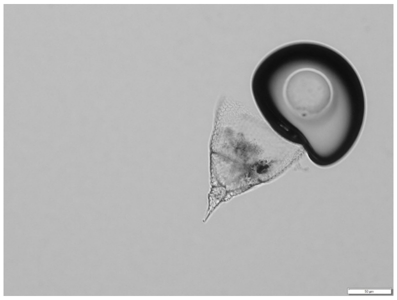

Specimen:
Pterocorys-zancleus-07

Order: Nassellaria

Cruise: HOT 339
Net tow#:3

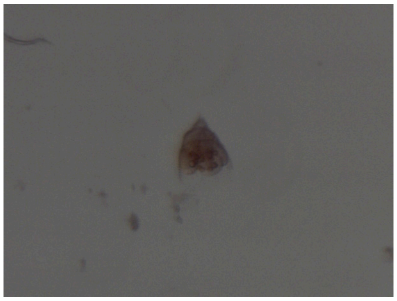

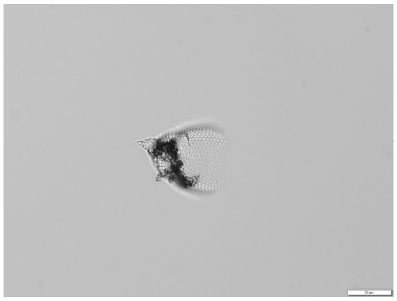

Specimen:
Pterocorys-zancleus-08

Order: Nassellaria

Cruise: HOT 339
Net tow#:13

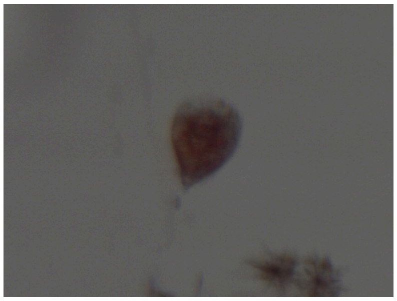

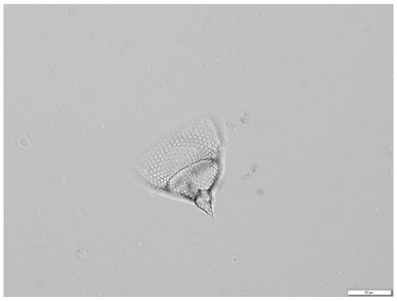

Specimen:
Pterocorys-zancleus-09

Order: Nassellaria

Cruise: HOT 339
Net tow#:13

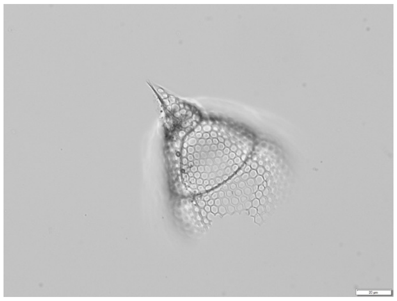

Specimen:
Pterocorys-zancleus-10

Order: Nassellaria

Cruise: HOT 338
Net tow#:9

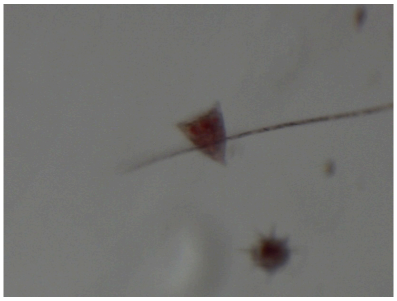

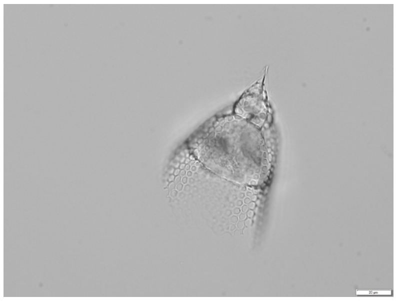

Specimen:
Pterocorys-zancleus-11

Order: Nassellaria

Cruise: HOT 339
Net tow#:14

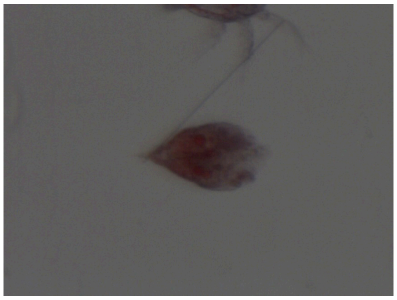

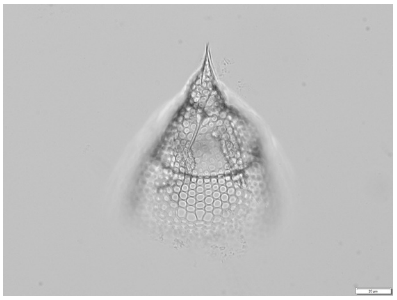

Specimen:
Pterocorys-zancleus-12

Order: Nassellaria

Cruise: HOT 339
Net tow#:14

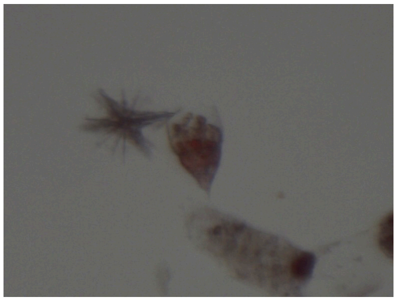

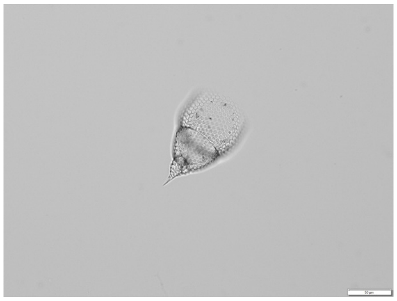

Specimen:
Pterocorys-zancleus-13

Order: Nassellaria

Cruise: HOT 339
Net tow#:14

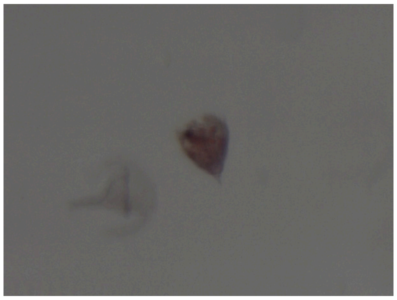

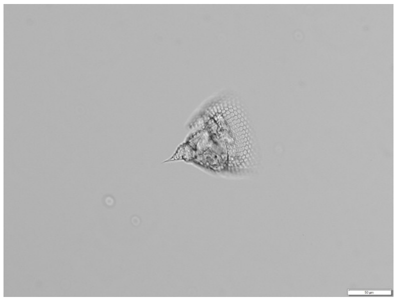

Specimen:
Pterocorys-zancleus-14

Order: Nassellaria

Cruise: HOT 339
Net tow#:14

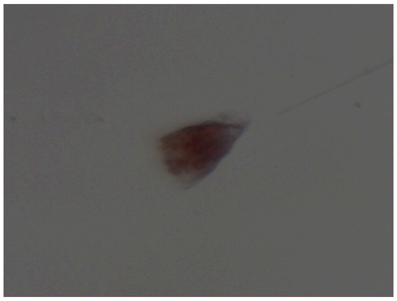

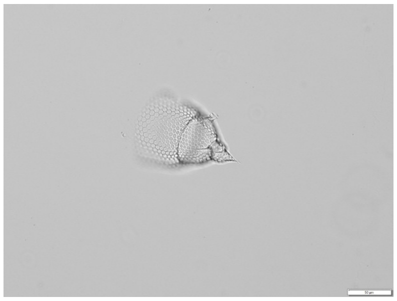

Specimen:
Pterocorys-zancleus-15

Order: Nassellaria

Cruise: HOT 339
Net tow#:18

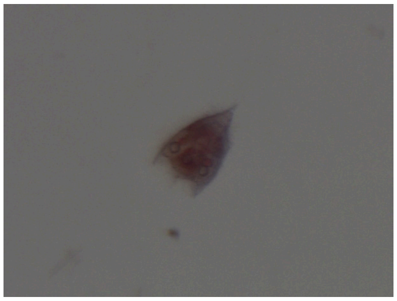

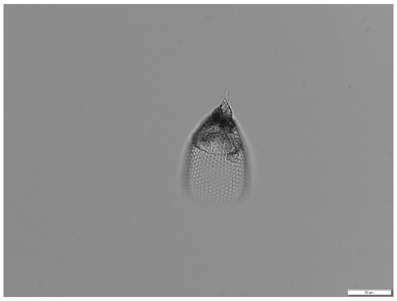

Specimen:
Pterocorys-zancleus-16

Order: Nassellaria

Cruise: HOT 339
Net tow#:19

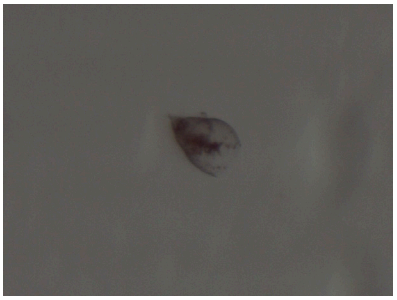

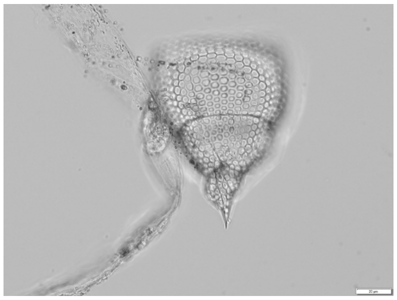

Specimen:
Pterocorys-zancleus-17

Order: Nassellaria

Cruise: HOT 339
Net tow#:19

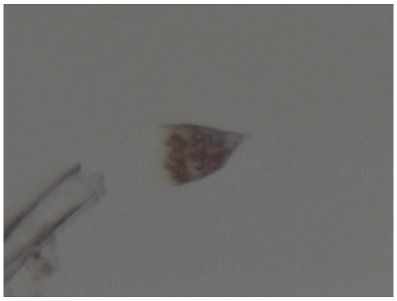

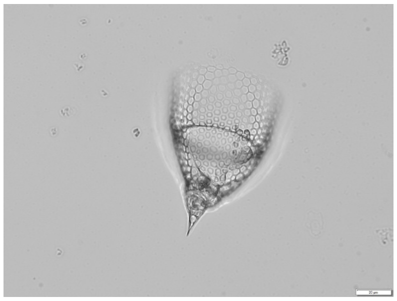

Specimen:
Pterocorys-zancleus-18

Order: Nassellaria

Cruise: HOT 339
Net tow#:19

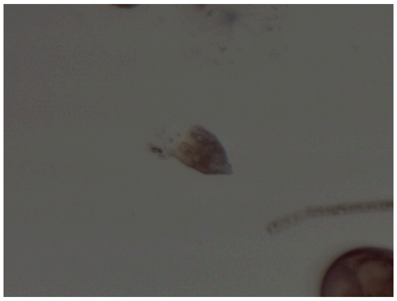
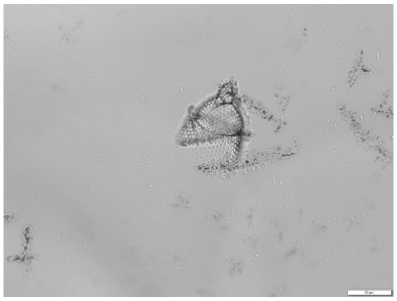

Specimen:
Pterocorys-zancleus-19

Order: Nassellaria

Cruise: HOT 339
Net tow#:8

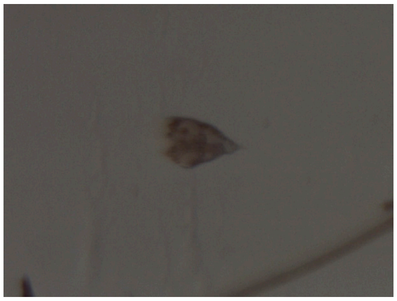
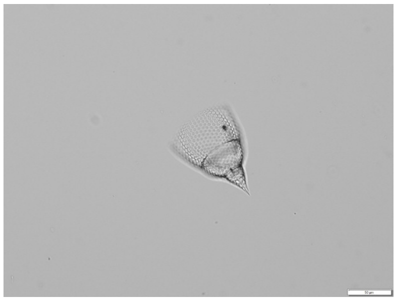

Specimen:
Tetracorethra-tetracorethra-01

Order: Nassellaria

Cruise: HOT 339
Net tow#:7

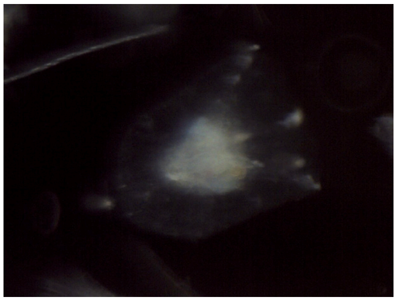
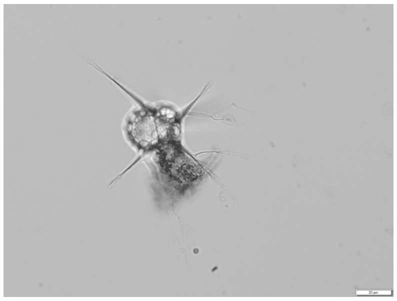

Specimen:
Pterocanium-praetextum-01

Order: Nassellaria

Cruise: HOT 339
Net tow#:10

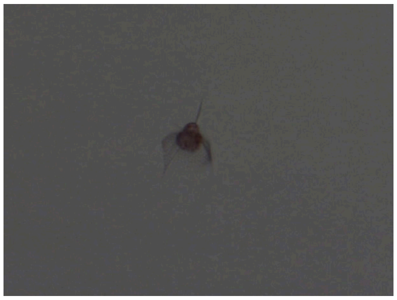
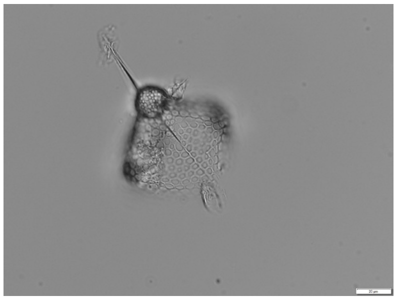

Specimen:
Pterocanium-praetextum-02

Order: Nassellaria

Cruise: HOT 339
Net tow#:7

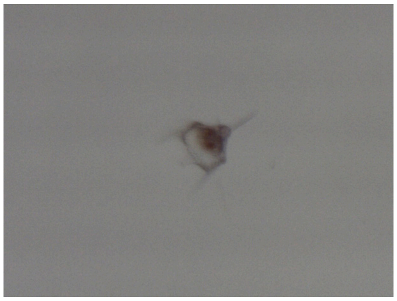
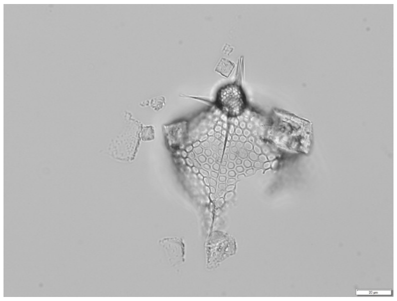

Specimen:
Pterocanium-praetextum-03

Order: Nassellaria

Cruise: HOT 339
Net tow#:9

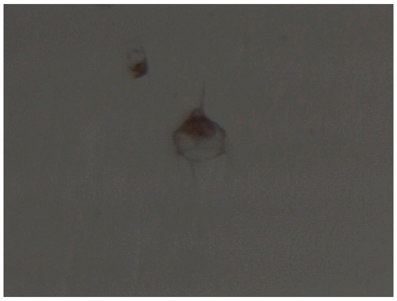
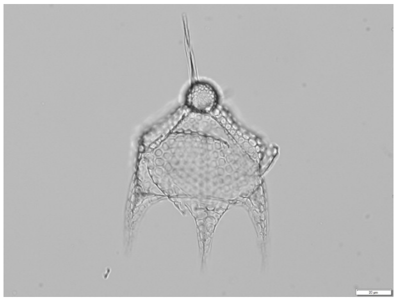

Specimen:
Dictyocoryne-profunda-01

Order: Spumellaria

Cruise: HOT 338
Net tow#:9

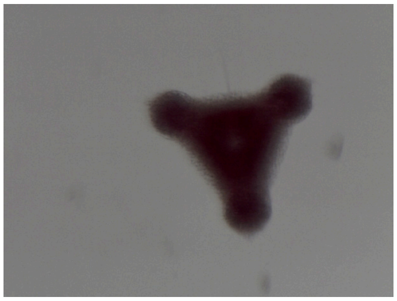
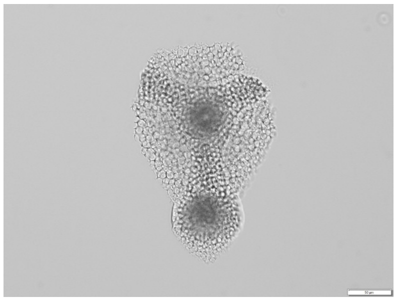

Specimen:
Dictyocoryne-profunda-02

Order: Spumellaria

Cruise: HOT 338
Net tow#:6

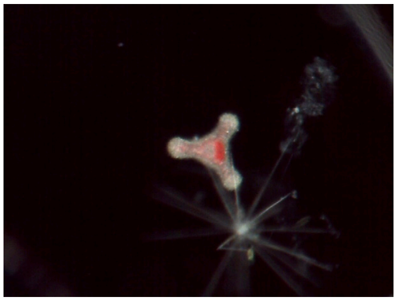
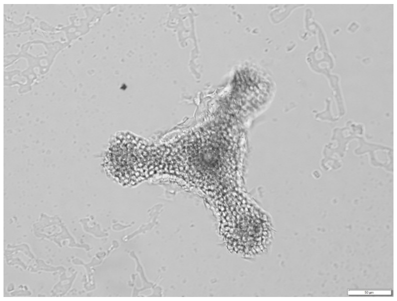

Specimen:
Dictyocoryne-profunda-03

Order: Spumellaria

Cruise: HOT 339
Net tow#:8

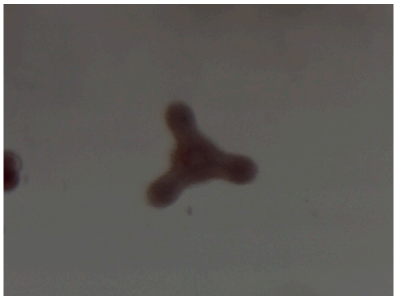
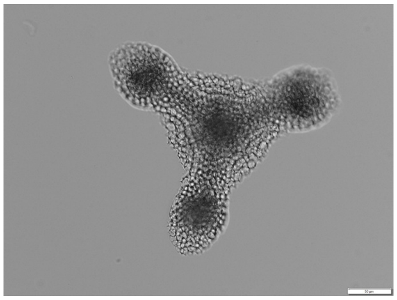

Specimen:
Dictyocoryne-profunda-04

Order: Spumellaria

Cruise: HOT 339
Net tow#:2

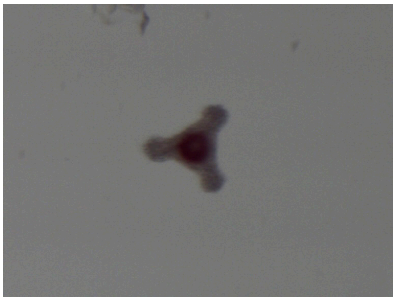
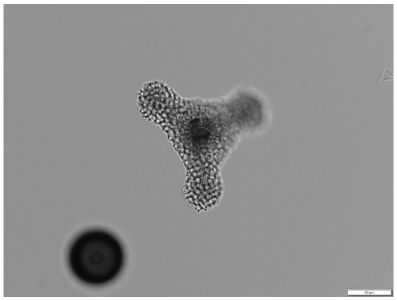

Specimen:
Dictyocoryne-profunda-05

Order: Spumellaria

Cruise: HOT 339
Net tow#:13

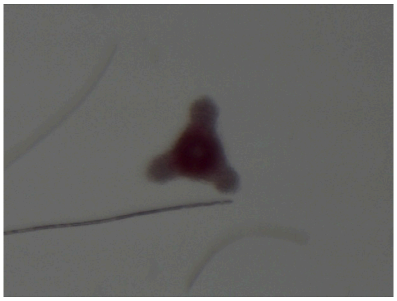

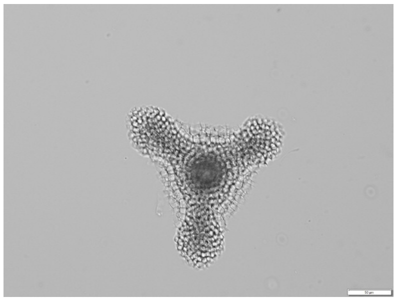

Specimen:
Dictyocoryne-profunda-06

Order: Spumellaria

Cruise: HOT 339
Net tow#:15

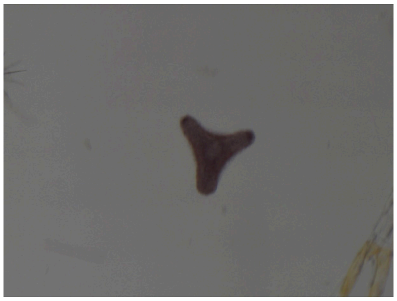

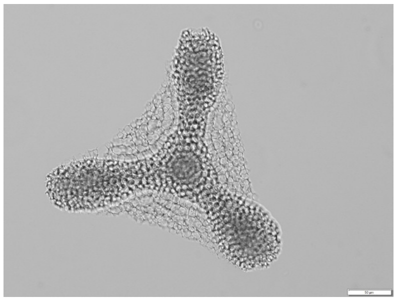

Specimen:
Dictyocoryne-sp-01

Order: Spumellaria

Cruise: HOT 338
Net tow#:9

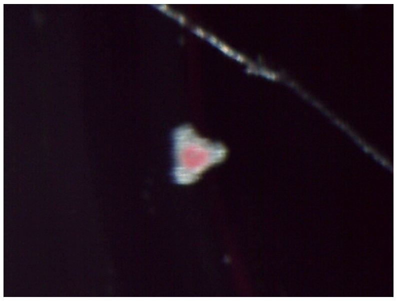

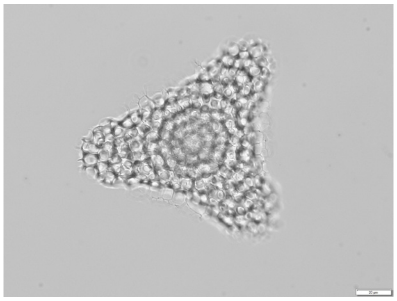

Specimen:
Dictyocoryne-sp-02

Order: Spumellaria

Cruise: HOT 339
Net tow#:10

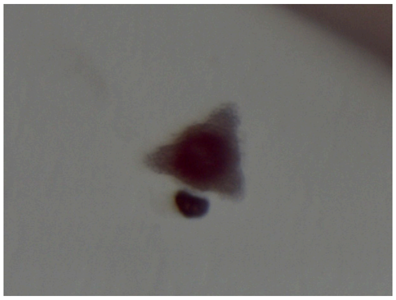

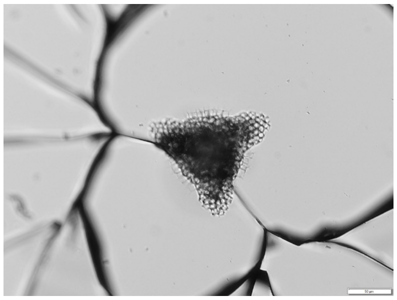

Specimen:
Dictyocoryne-sp-03

Order: Spumellaria

Cruise: HOT 339
Net tow#:13

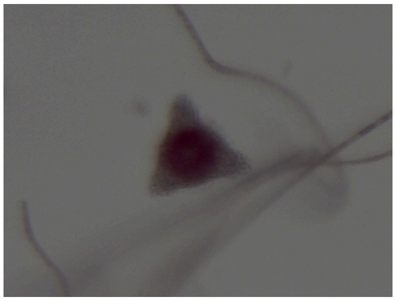

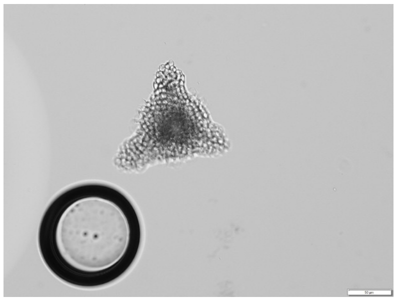

Specimen:
Dictyocoryne-sp-04

Order: Spumellaria

Cruise: HOT 339
Net tow#:2

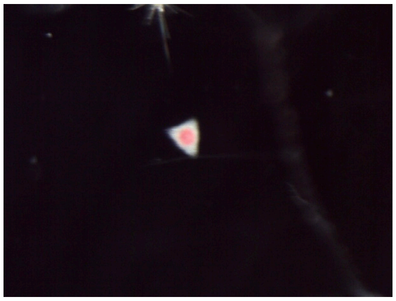

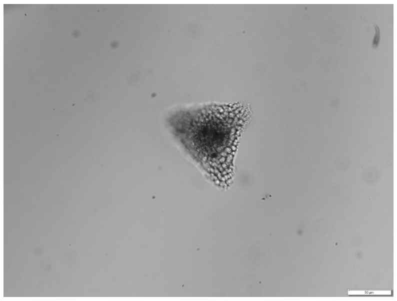

Specimen:
Dictyocoryne-truncatum-01

Order: Spumellaria

Cruise: HOT 339
Net tow#:15

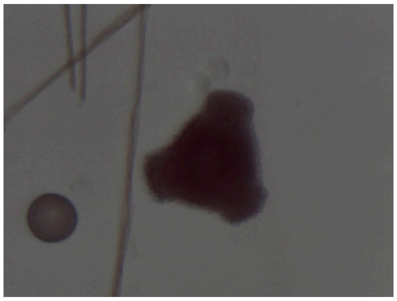

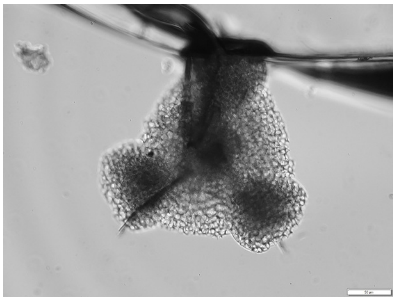

Specimen:
Dictyocoryne-truncatum-02

Order: Spumellaria

Cruise: HOT 339
Net tow#:8

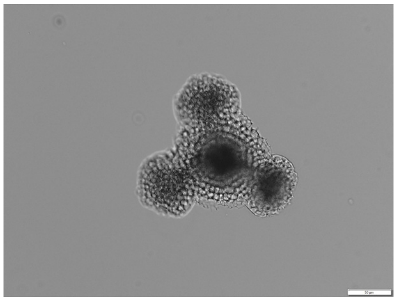

Specimen:
Dictyocoryne-truncatum-03

Order: Spumellaria

Cruise: HOT 339
Net tow#:19

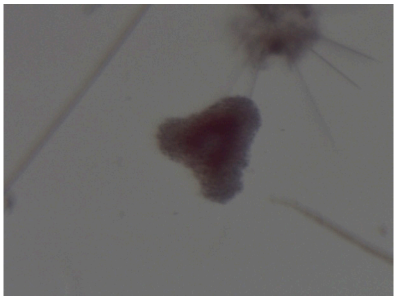

Specimen:
Didymocyrtis-sp-01

Order: Spumellaria

Cruise: HOT 339
Net tow#:15

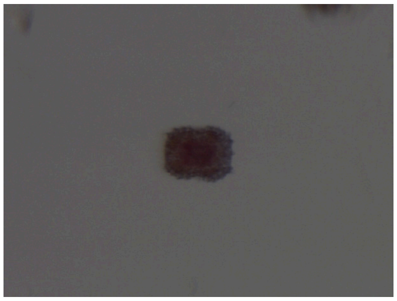

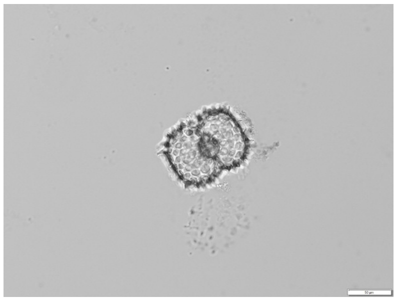

Specimen:
Didymocyrtis-sp-02

Order: Spumellaria

Cruise: HOT 339
Net tow#:10

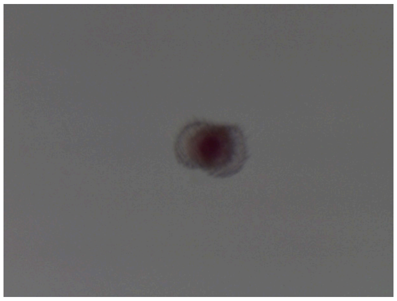

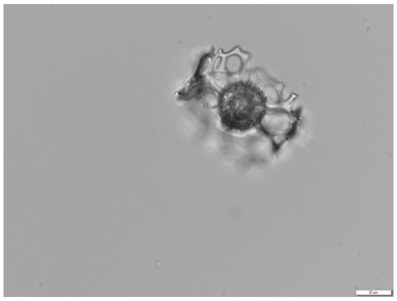

Specimen:
Didymocyrtis-sp-03

Order: Spumellaria

Cruise: HOT 339
Net tow#:15

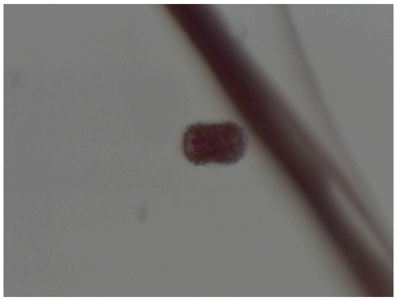

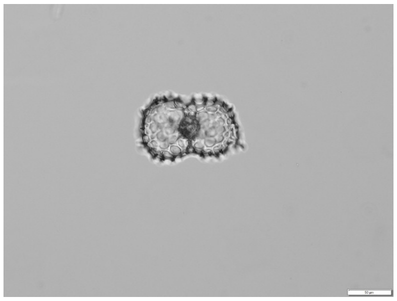

Specimen:
Didymocyrtis-sp-04

Order: Spumellaria

Cruise: HOT 339
Net tow#:15

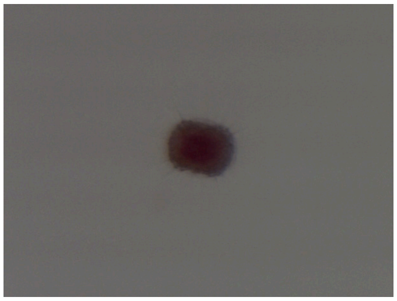

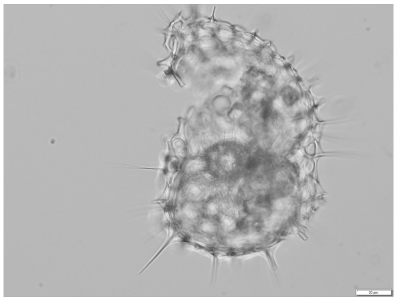

Specimen:
Didymocyrtis-sp-05

Order: Spumellaria

Cruise: HOT 339
Net tow#:3

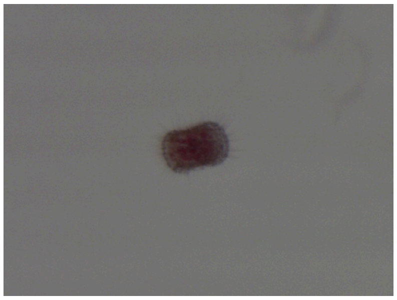

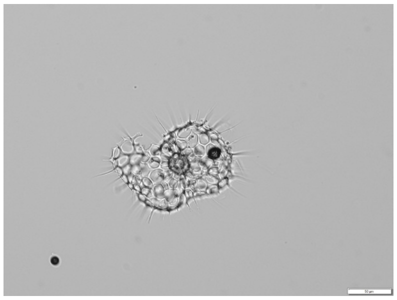

Specimen:
Didymocyrtis-sp-06

Order: Spumellaria

Cruise: HOT 339
Net tow#:7

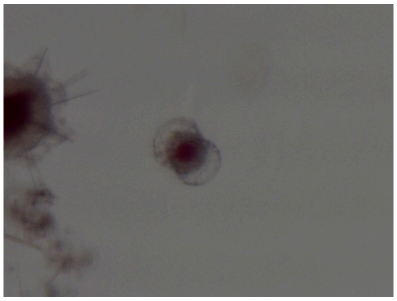

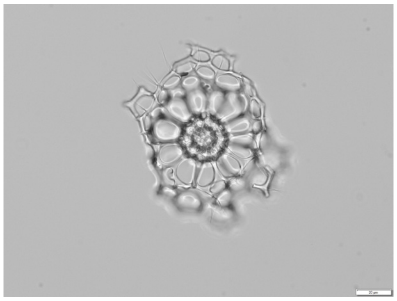

Specimen:
Didymocyrtis-tetrathalamus-coronatus-01

Order: Spumellaria

Cruise: HOT 339
Net tow#:13

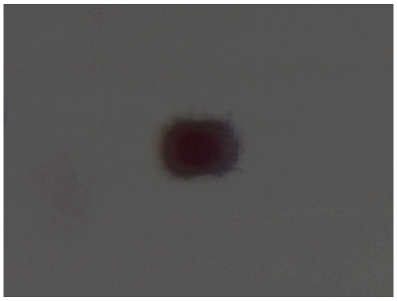

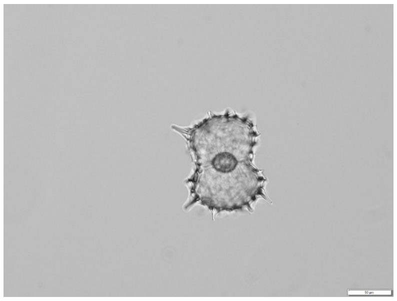

Specimen:
Didymocyrtis-tetrathalamus-coronatus-02

Order: Spumellaria

Cruise: HOT 338
Net tow#:9

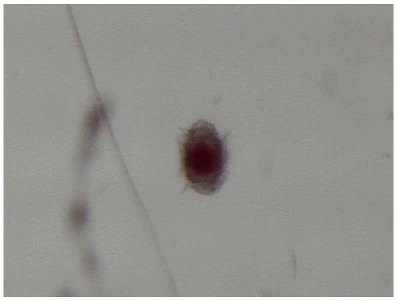

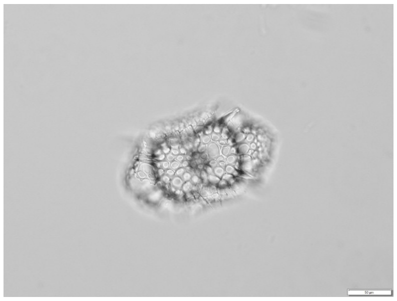

Specimen:
Didymocyrtis-tetrathalamus-coronatus-03

Order: Spumellaria

Cruise: HOT 338
Net tow#:9

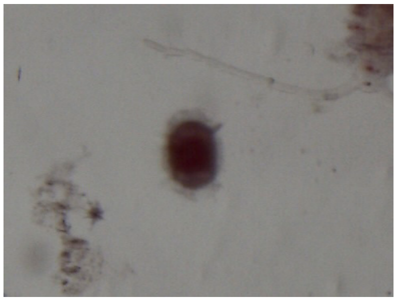

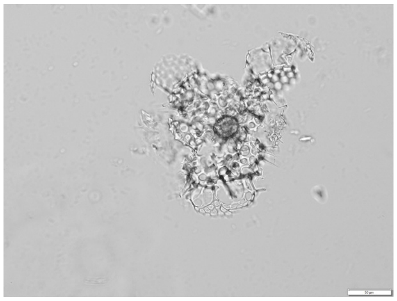

Specimen:
Didymocyrtis-tetrathalamus-tetrathalamus-01

Order: Spumellaria

Cruise: HOT 339
Net tow#:13

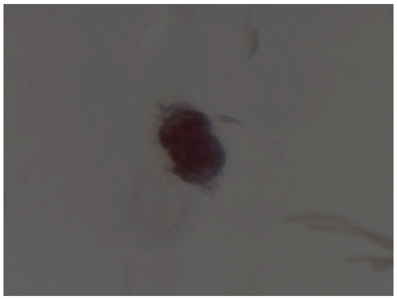

Specimen:
Didymocyrtis-tetrathalamus-tetrathalamus-02

Order: Spumellaria

Cruise: HOT 339
Net tow#:13

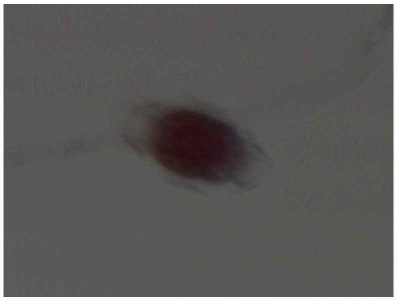

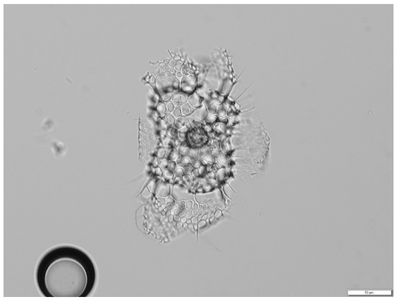

Specimen:
Didymocyrtis-tetrathalamus-tetrathalamus-03

Order: Spumellaria

Cruise: HOT 339
Net tow#:15

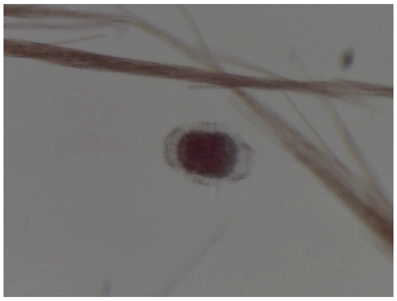

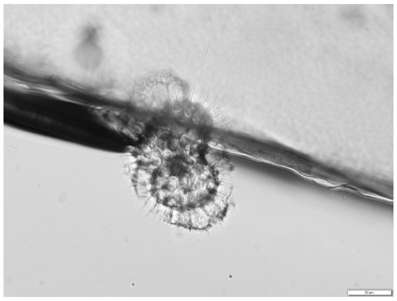

Specimen:
Didymocyrtis-tetrathalamus-tetrathalamus-04

Order: Spumellaria

Cruise: HOT 339
Net tow#:15

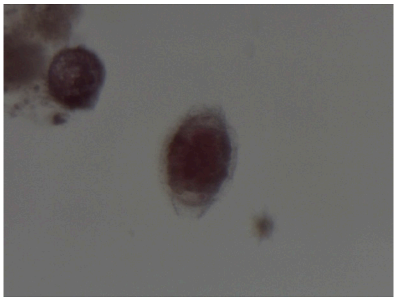

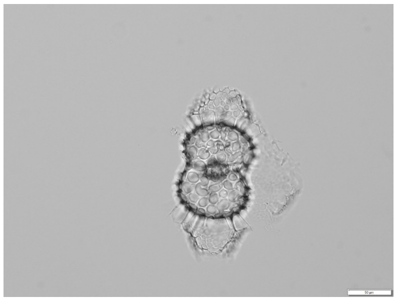

Specimen:
Didymocyrtis-tetrathalamus-tetrathalamus-05

Order: Spumellaria

Cruise: HOT 339
Net tow#:2

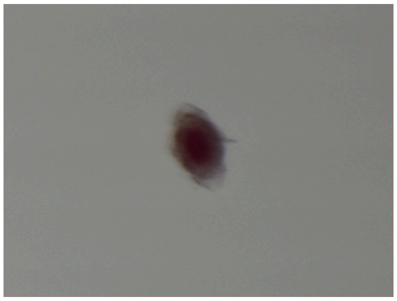

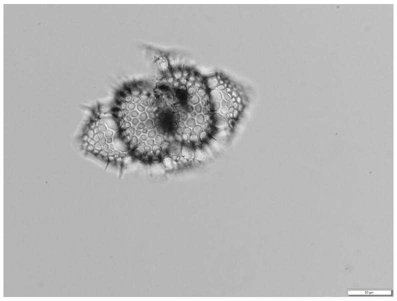

Specimen:
Didymocyrtis-tetrathalamus-tetrathalamus-06

Order: Spumellaria

Cruise: HOT 338
Net tow#:7

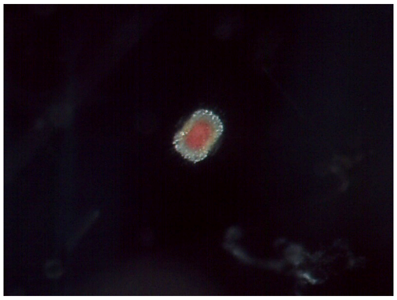

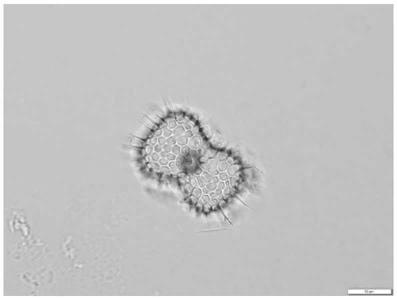

Specimen:
Didymocyrtis-tetrathalamus-tetrathalamus-07

Order: Spumellaria

Cruise: HOT 338
Net tow#:9

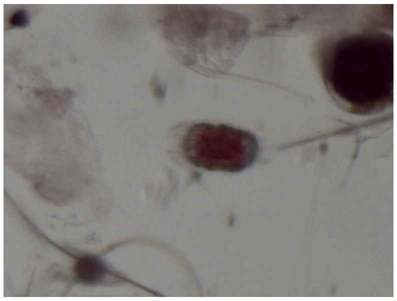

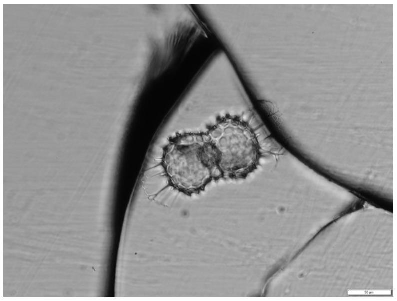

Specimen:
Euchitonia-elegans-furcata-01

Order: Spumellaria

Cruise: HOT 338
Net tow#:3

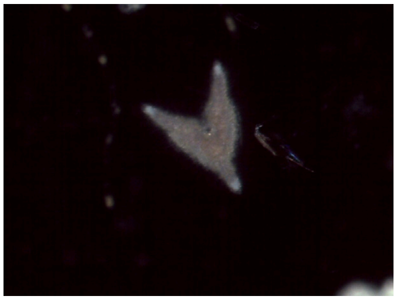
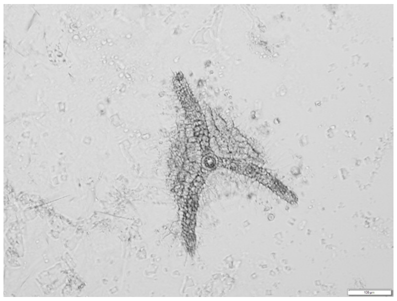

Specimen:
Euchitonia-elegans-furcata-02

Order: Spumellaria

Cruise: HOT 338
Net tow#:4

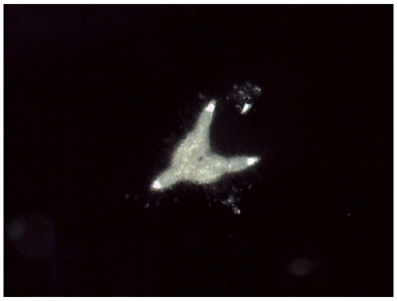
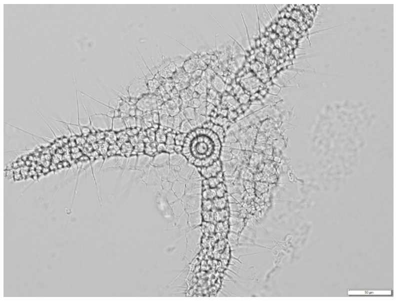

Specimen:
Euchitonia-elegans-furcata-03

Order: Spumellaria

Cruise: HOT 338
Net tow#:8

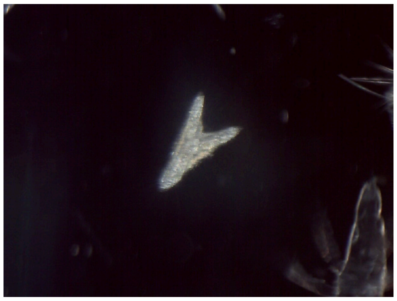
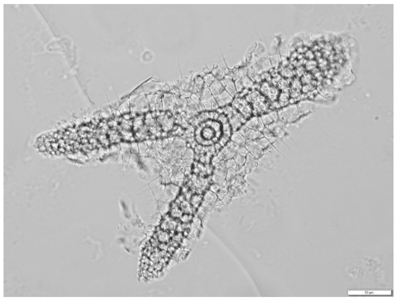

Specimen:
Euchitonia-elegans-furcata-04

Order: Spumellaria

Cruise: HOT 338
Net tow#:9

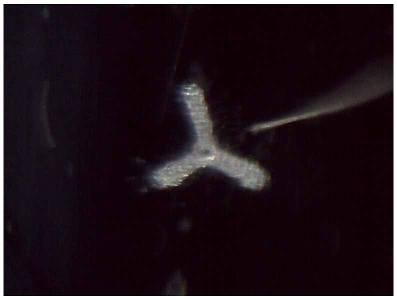
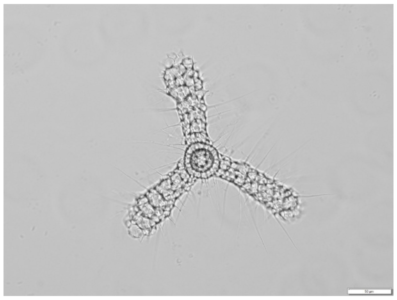

Specimen:
Euchitonia-elegans-furcata-05

Order: Spumellaria

Cruise: HOT 339
Net tow#:13

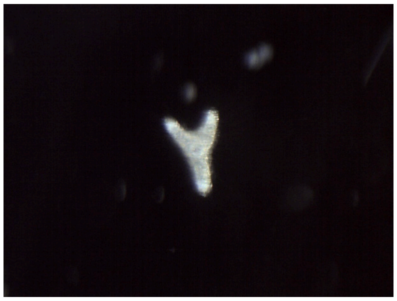
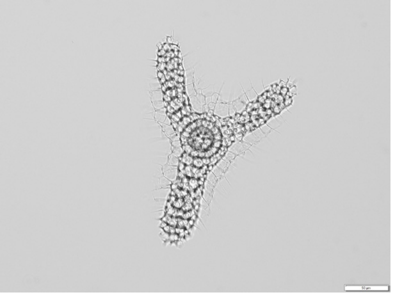

Specimen:
juvenile-Pyloniidae-sp-01

Order: Spumellaria

Cruise: HOT 339
Net tow#:6

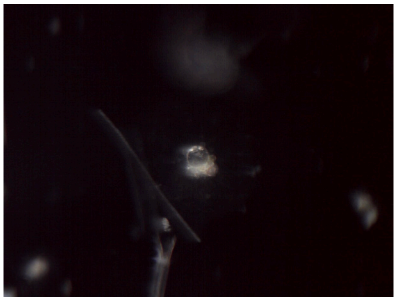

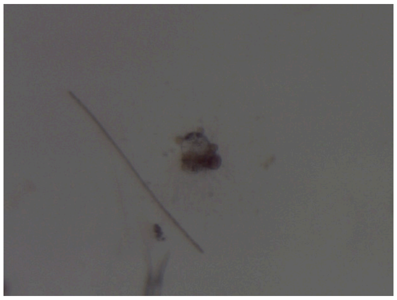

Specimen:
juvenile-Spumellaria-01

Order: Spumellaria

Cruise: HOT 339
Net tow#:10

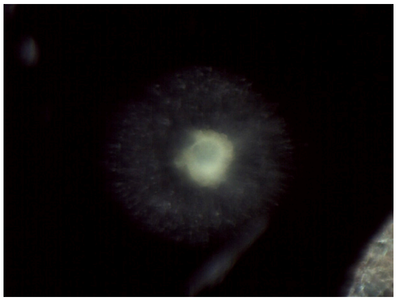

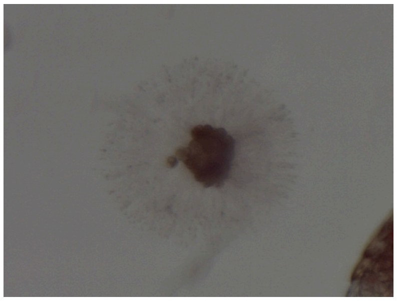

Specimen:
juvenile-Spumellaria-02

Order: Spumellaria

Cruise: HOT 339
Net tow#:18

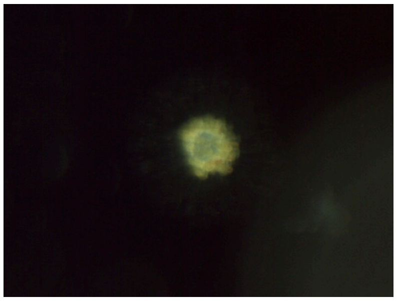

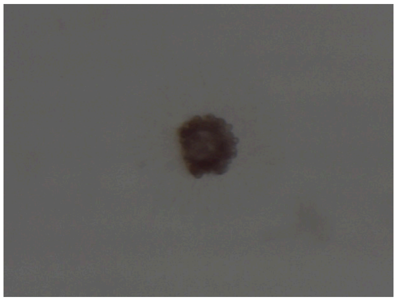

Specimen:
lithellid-sp3-01

Order: Spumellaria

Cruise: HOT 338
Net tow#:9

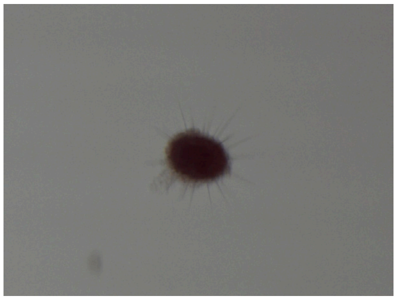

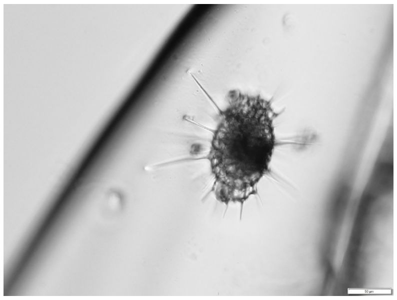

Specimen:
Pyloniidae-sp-01

Order: Spumellaria

Cruise: HOT 339
Net tow#:10

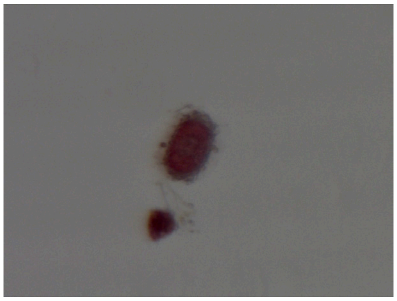

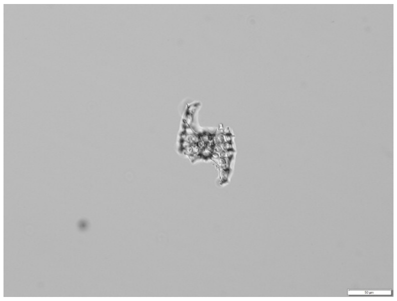

Specimen:
Pyloniidae-sp-02

Order: Spumellaria

Cruise: HOT 339
Net tow#:10

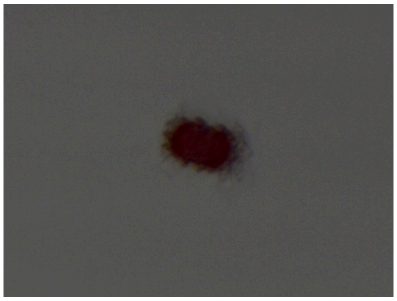

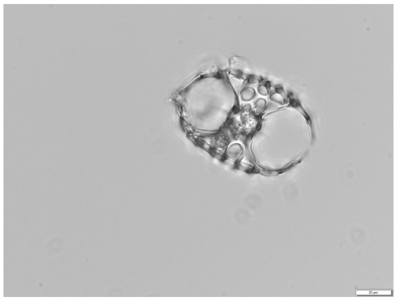

Specimen:
Pyloniidae-sp-03

Order: Spumellaria

Cruise: HOT 339
Net tow#:19

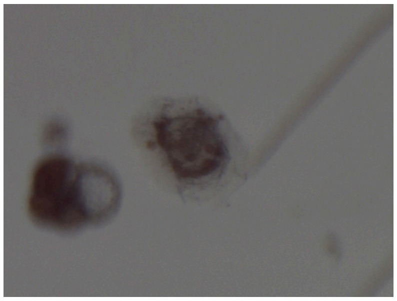

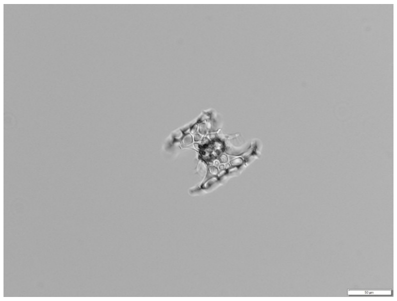

Specimen:
Pyloniidae-sp-04

Order: Spumellaria

Cruise: HOT 339
Net tow#:19

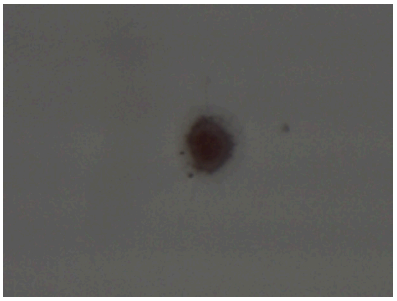

Supplement: S1 Fig — Photos taken of live specimens in darkfield and brightfield with the shipboard dissecting scope have been cropped and adjusted for white balance and contrast when necessary. Photos taken of skeletal remains with the compound microscope are unedited. Specimens collected on the SCOPE-PARAGON II cruise were not photographed live, so only photos of skeletal remains are included. Other specimens that only have one photo either could not be photographed on board the ship due to high rocking motion, or they broke beyond recognition during/after DNA extraction and could only be identified from their live photos. (PDF) [file pone.0326053.s001.pdf]
