# Supplementary figures and images for: Genetic variability in microbial eukaryotes reshapes marine biodiversity assessment in the age of amplicon sequencing
Source: PLoS One. 2025 Jun 20;20(6):e0326053. doi: 10.1371/journal.pone.0326053 (PMC12180732; doi:10.1371/journal.pone.0326053)

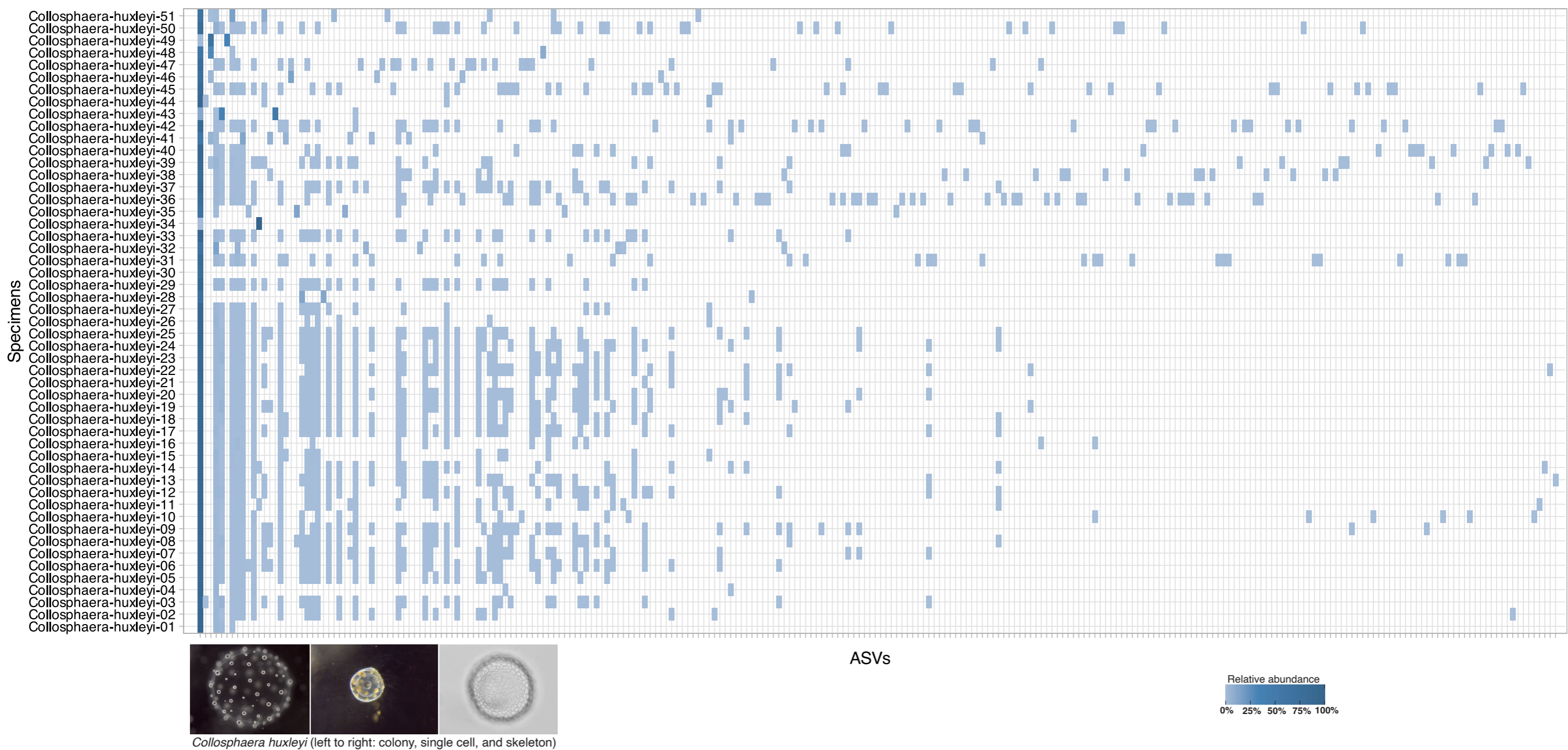

Supplement: S2 Fig — Specimen names are shown on the y-axis, corresponding to specimen names in S1 Data and S2 Fig. The x-axis denotes individual ASVs (represented by tick marks) present in each specimen of the morphospecies. Relative read abundances of ASVs per specimen are indicated by the shade of blue (light = low abundance, dark = high abundance); ASVs not present in a particular specimen are indicated as white background. Representative micrographs of C. huxleyi are shown below the heatmap. (PDF) [file pone.0326053.s002.pdf]

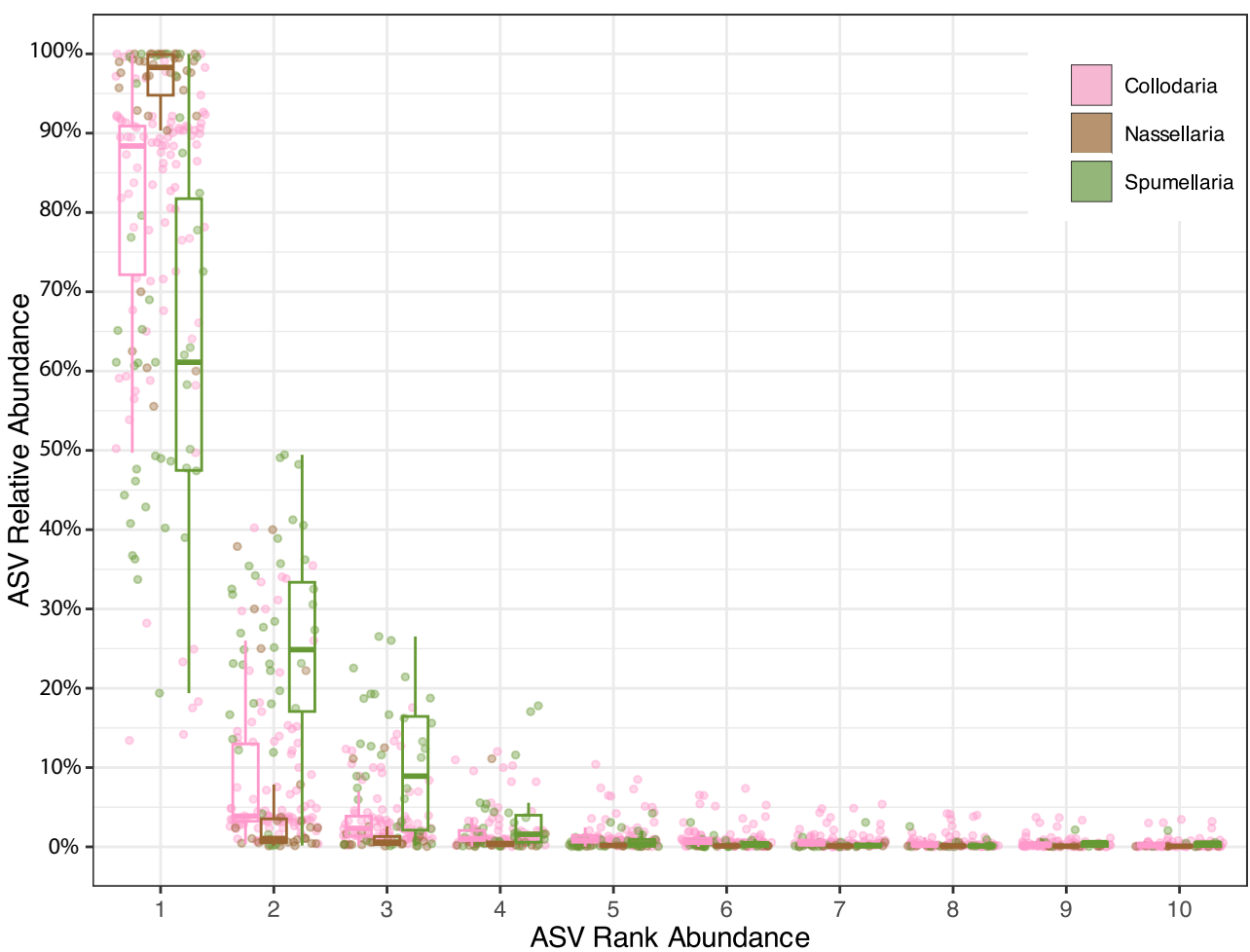

Supplement: S3 Fig — Dots represent relative V4-ASV abundances (percent of total reads yielded by each specimen) at each designated rank (1st–10th) for all 173 specimens. Boxplots denote the median, interquartile range, maximum, and minimum values (outliers excluded) of V4-ASV relative abundances for each order (Collodaria = pink; Nassellaria = brown; Spumellaria = green). Data underlying this figure is in S1 Data. (PDF) [file pone.0326053.s003.pdf]

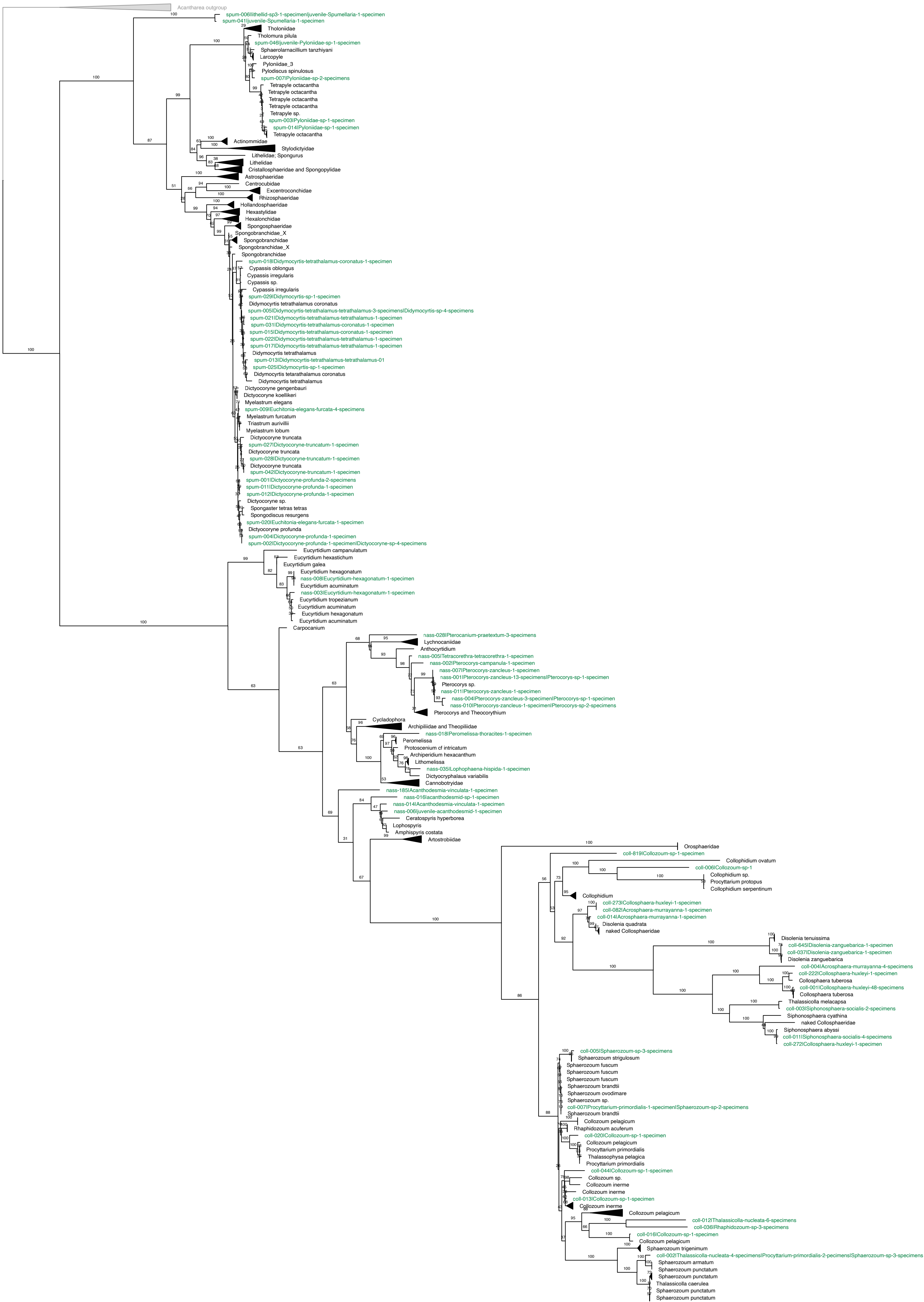

Supplement: S4 Fig — Tip labels indicate the morphological taxonomy of specimens with a given dominant ASV, as well as the number of specimens that shared the same dominant ASV. ASV identifiers correspond with those in S1 Data. Polycystine radiolarian reference sequences downloaded from PR2 are denoted with black text. Green text indicates new ASVs obtained from specimens in this study. Parameters used to generate this tree are described in “Materials and methods.” Accession numbers for the polycystine reference sequences and acantharian outgroup sequences can be found in S1 Table. (PDF) [file pone.0326053.s004.pdf]

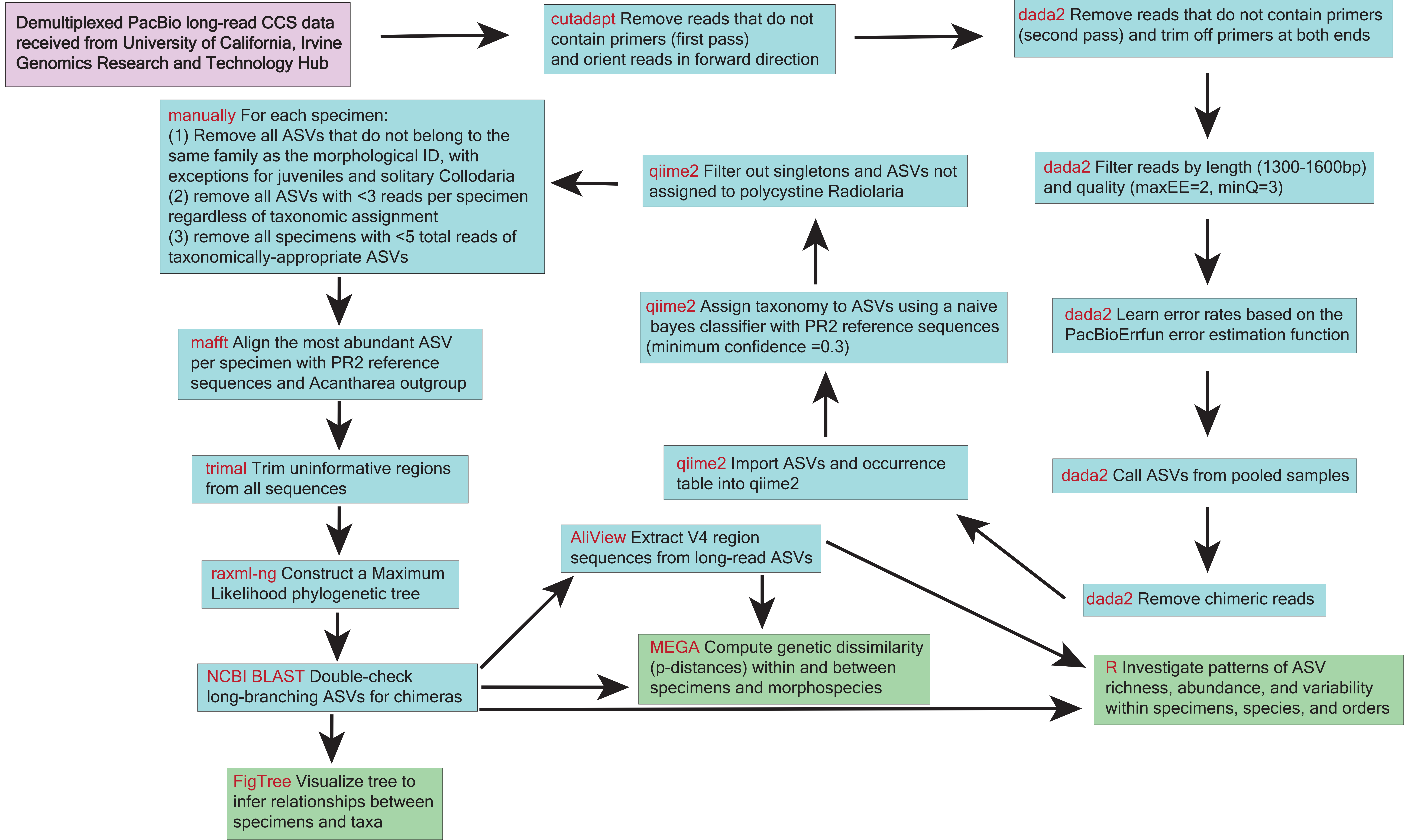

Supplement: S6 Fig — The purple box denotes the type of input data, blue boxes show intermediate steps in the workflow, and green boxes indicate the main results discussed in this study. (PDF) [file pone.0326053.s006.pdf]
