# Supplementary material for: Genetic variability in microbial eukaryotes reshapes marine biodiversity assessment in the age of amplicon sequencing
Source: PLoS One. 2025 Jun 20;20(6):e0326053. doi: 10.1371/journal.pone.0326053 (PMC12180732; doi:10.1371/journal.pone.0326053)
Supplement: S5 Fig — Each box displays specimens that shared dominant ASVs and are presumably intraspecific, and/or have a dominant ASV that closely matches a reference sequence in PR2 or GenBank. Background colors indicate the polycystine order determined based on ASV taxonomic assignments (pink = Collodaria; brown = Nassellaria; green = Spumellaria). These solitary specimens lacked any siliceous skeletonization needed for morphological taxonomic classification, but molecular sequencing revealed taxonomic identities in all three orders. This morphotype is therefore hypothesized to be a previously-undescribed juvenile life stage that has similar characteristics in Collodaria, Nassellaria, and Spumellaria. (PDF) [file pone.0326053.s005.pdf]

(a)

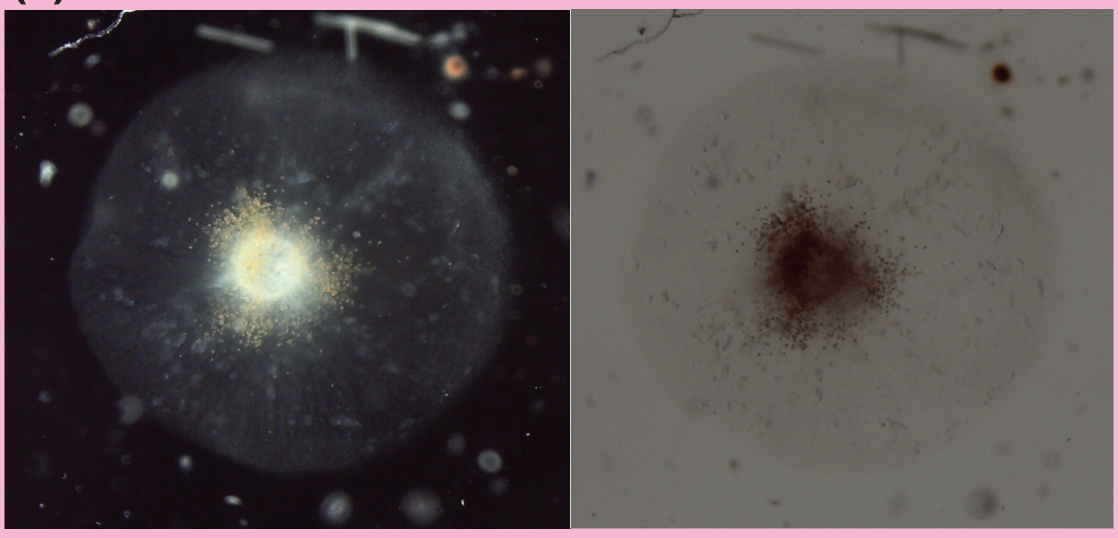

Specimen id: Procyttarium-primordialis?-02
Dominant ASV: coll-002
Best match: *Sphaerozoum armatum* with 77% confidence in PR2 and 99% identity in GenBank.
Notes: The dominant ASV in this specimen was shared by other specimens morphologically identified as *Procyttarium primordialis?*, *Thalassicolla nucleata*, and *Sphaerozoum* sp. All of these morphotaxa may be different life stages of the same species.

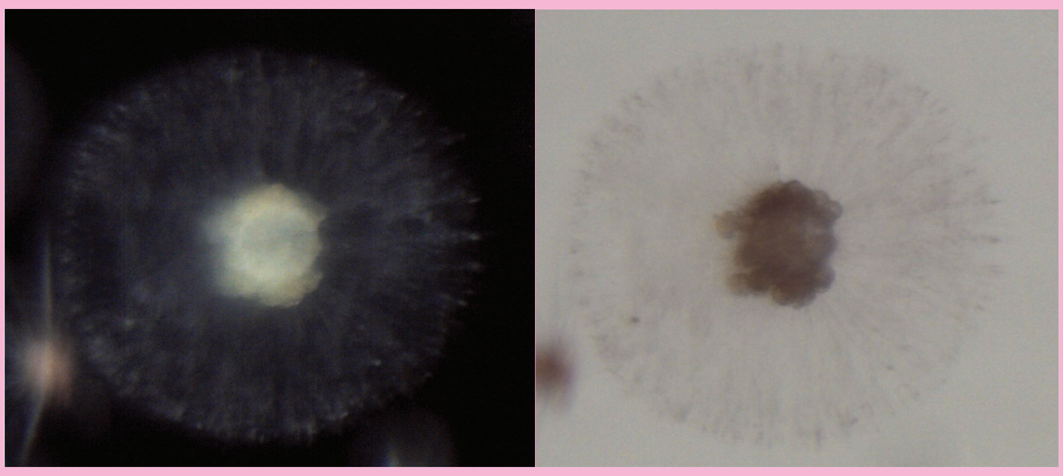

Specimen id: Procyttarium-primordialis?-03
Dominant ASV: coll-002
Best match: *Sphaerozoum armatum* with 77% confidence in PR2 and 99% identity in GenBank.
Notes: Same as left.

(b)

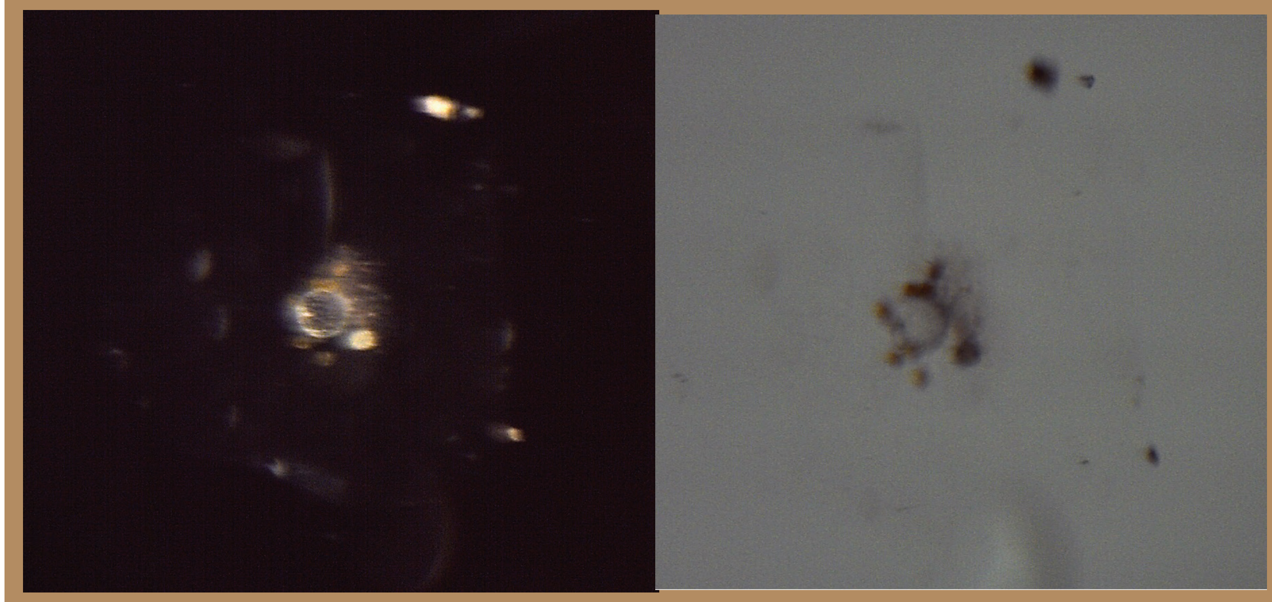

Specimen id: juvenile-acanthodesmid-01
Dominant ASV: nass-006
Best match: PR2 reference for *Ceratospyris hyperborea* (Nassellaria; Acanthodesmidae) with 99.9% confidence.

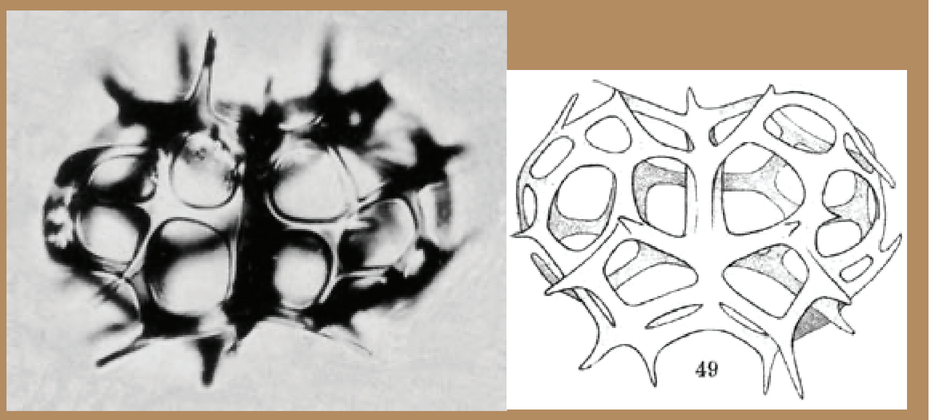

Ceratospyris hyperborea Jorgenson (1905)
Line drawing of holotype from Jorgenson (1905).
Photograph by Kjell Bjørklund depicting probable syntype from Jorgenson's slide collection.

(c)

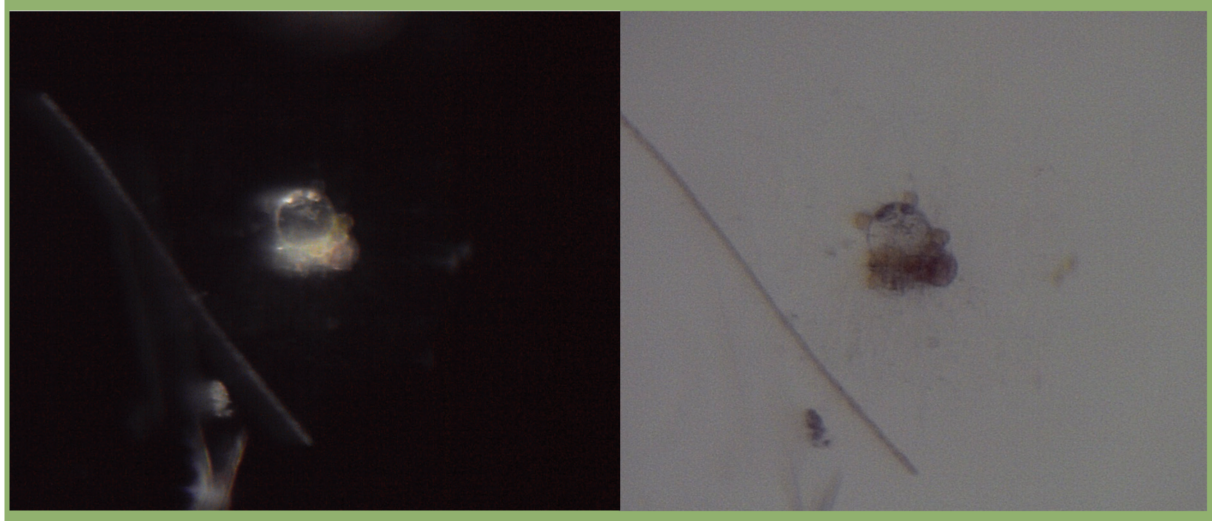

Specimen id: juvenile-Pyloniidae-01
Dominant ASV: spum-046
Best match: *Larcopyle butschlii* (Spumellaria) with 99.2% identity in GenBank, and with 80% confidence in PR2.

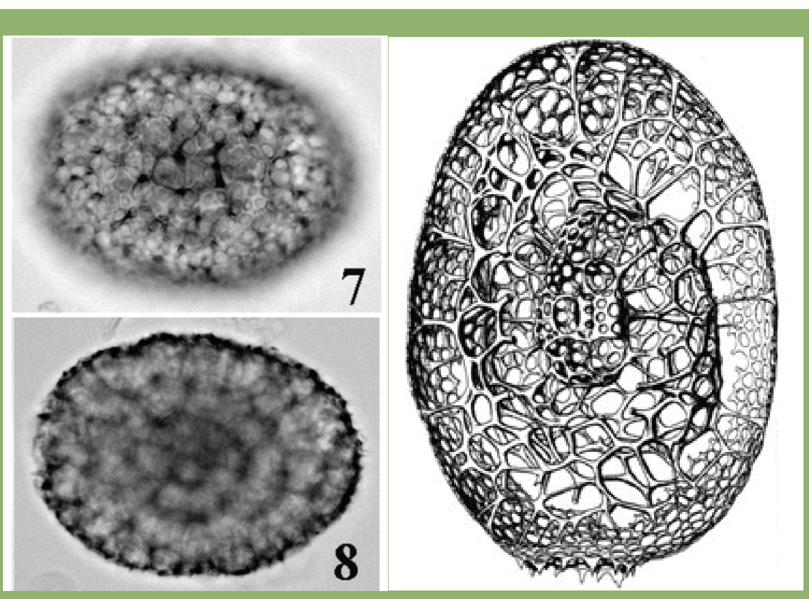

Larcopyle buetschlii Dyer (1889)
Line drawing of holotype from Dyer (1889).
Photographs of topotype by Zhang and Suzuki (2017).

(d)

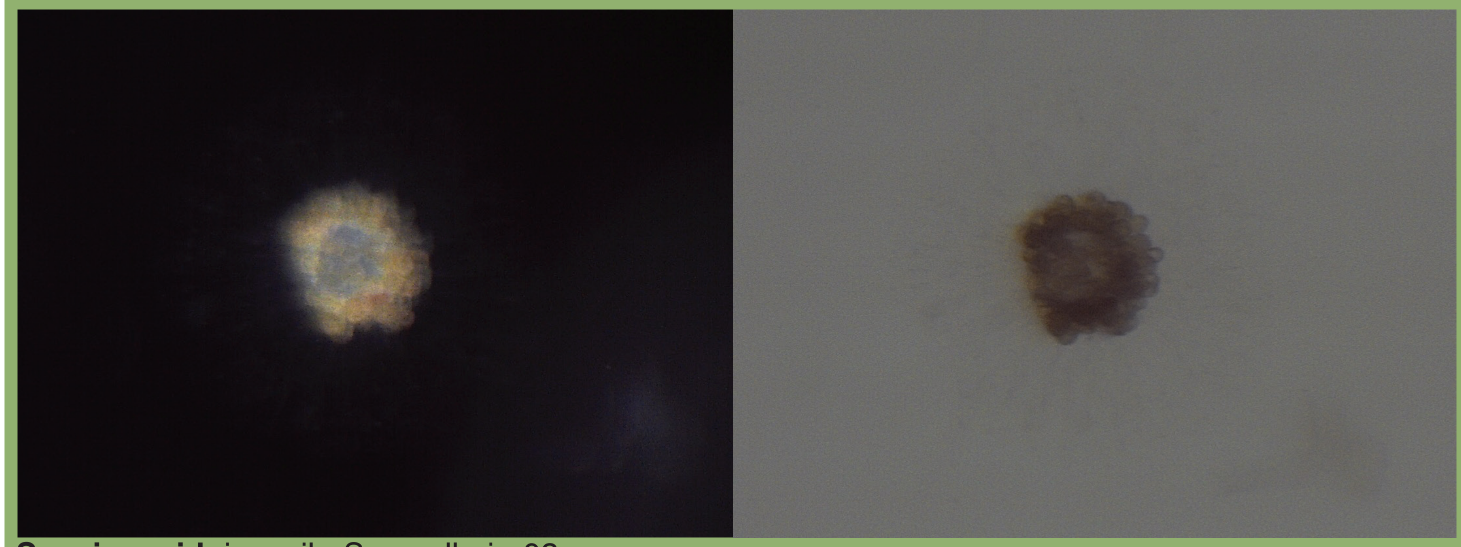

Specimen id: juvenile-Spumellaria-02
Dominant ASV: spum-006
Best match: order Spumellaria with 99.4% confidence in PR2; no close matches in PR2 or GenBank to lower taxonomic levels.
Notes: This specimen shared its dominant ASV and multiple subdominant ASVs with another spumellarian specimen, lithellid-sp-3-01. This specimen also contained the ASV spum-041, which was dominant in the specimen juvenile-Spumellaria-01 (below), suggesting all three specimens may be closely related or intraspecific.

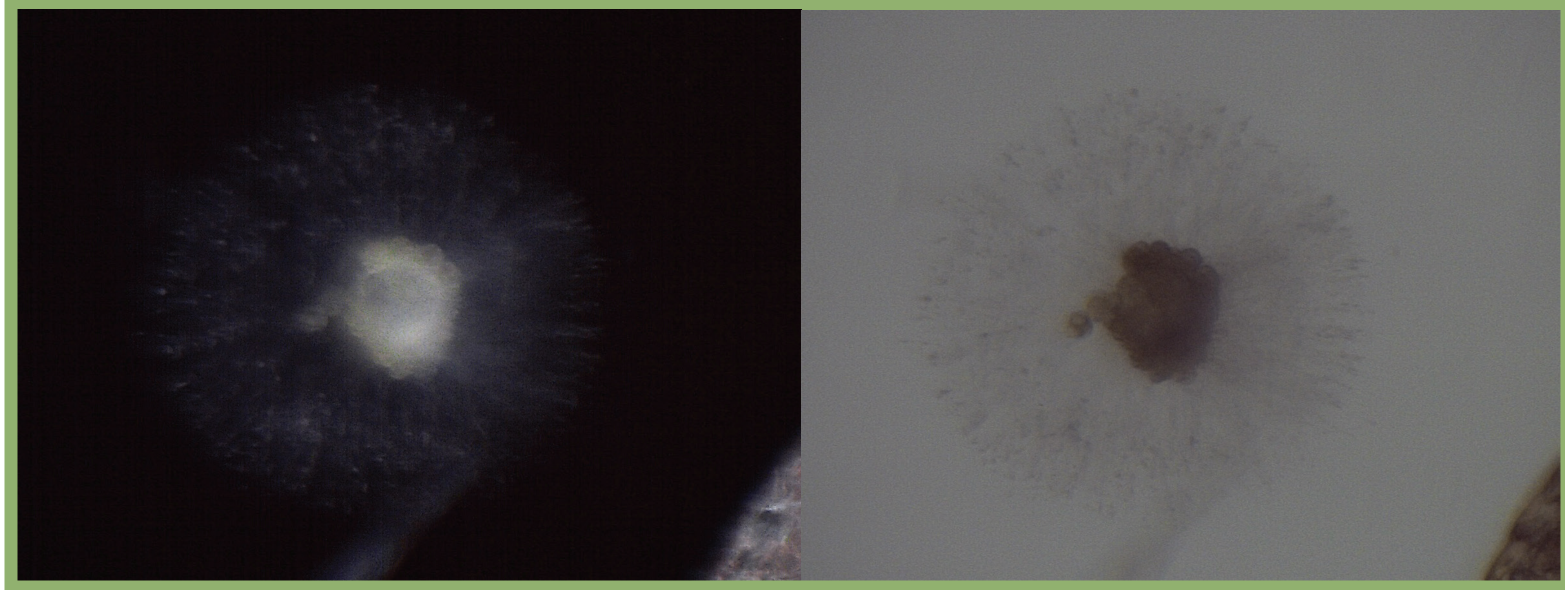

Specimen id: juvenile-Spumellaria-01
Dominant ASV: spum-041
Best match: order Spumellaria with 99.1% confidence in PR2; no close matches in PR2 or GenBank to lower taxonomic levels.
Notes: This specimen's 2nd ranking ASV was spum-006 (211 reads), suggesting that it may be closely related to or intraspecific with the specimens juvenile-Spumellaria-02 (above) and lithellid-sp-3-01 (right).

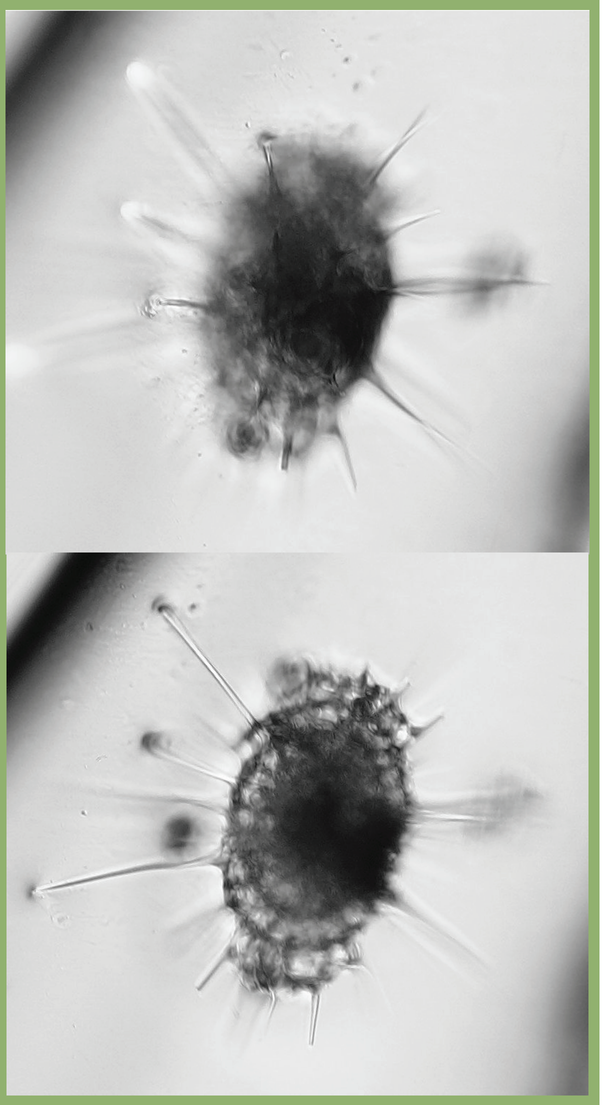

Specimen id: lithellid-sp-3-01
Dominant ASV: spum-006
Best match: order Spumellaria with 99.4% confidence in PR2; no close matches in PR2 or GenBank to lower taxonomic levels.
Notes: This specimen was morphologically identified as an open nomenclature taxon illustrated in Trubovitz et al. (2020): unknown lithellid sp. 3. This specimen shared its dominant and subdominant ASVs with two putative juvenile specimens (left), which lacked any skeletal development.
